# Supplementary material for: CaMKII nucleates an osmotic protein supercomplex to induce cellular bleb expansion
Source: EMBO J. 2026 Feb 3;45(8):2433–55. doi: 10.1038/s44318-026-00703-5 (PMC13083957; doi:10.1038/s44318-026-00703-5)
Supplement: Supplementary file 1 — Appendix [file 44318_2026_703_MOESM1_ESM.pdf]

Appendix for

CaMKII nucleates an osmotic protein supercomplex to induce cellular bleb expansion

Table of contents

Appendix Figure S1	1
Appendix Figure S2	2
Appendix Figure S3	3
Appendix Figure S4	4～5
Appendix Table S1	6～7

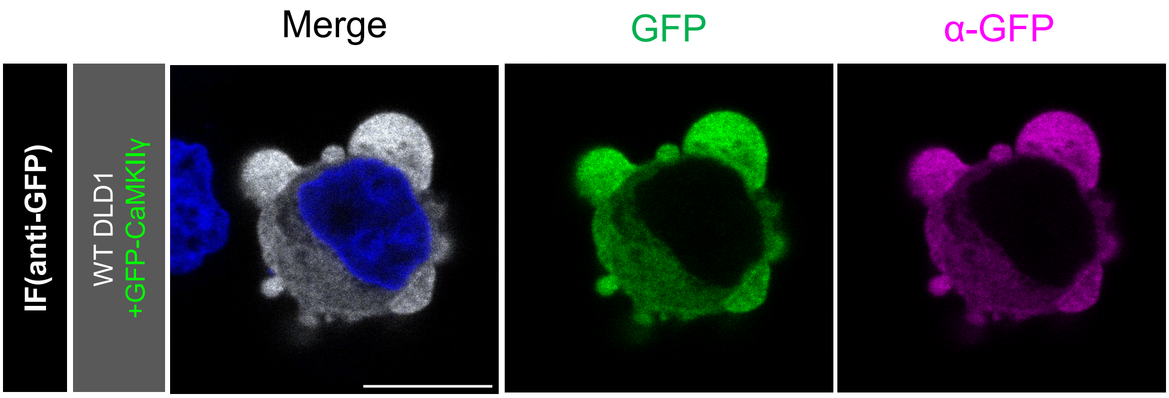

Appendix Figure S1

Increased GFP-CaMKII fluorescence intensity within expanding blebs reflects the accumulation of GFP-CaMKII

Immunofluorescence image of DLD1 cells expressing GFP–CaMKIIγ, stained with an anti-GFP antibody. (Scale bar: 10 μm)

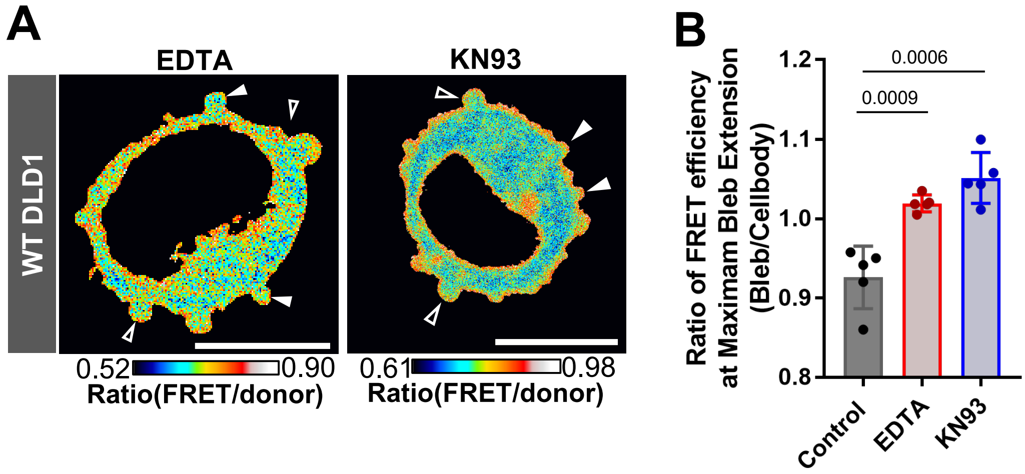

Appendix Figure S2

In cells treated with EDTA or KN-93, CaMKII fails to adopt an activation-competent extended conformation within expanding blebs

(A) FRET/donor ratio images of Camui-CR–expressing WT cells treated with the indicated reagents.

(B) FRET efficiency ratio between bleb and cell body regions at the point of maximal bleb expansion in Camui-CR–expressing WT cells treated with the indicated reagents. Under control conditions, FRET efficiency was lower in blebs than in the adjacent cell body regions, whereas this bleb-specific decrease was not observed in cells treated with EDTA or KN93. For each condition, five blebs were analyzed, and the ratio between bleb and cell body regions was calculated. Statistical significance was assessed using Student's t-test; exact p values are $p = 0.0009$ (for Ctrl vs. EDTA), $p = 0.0006$ (for Ctrl vs. KN93).

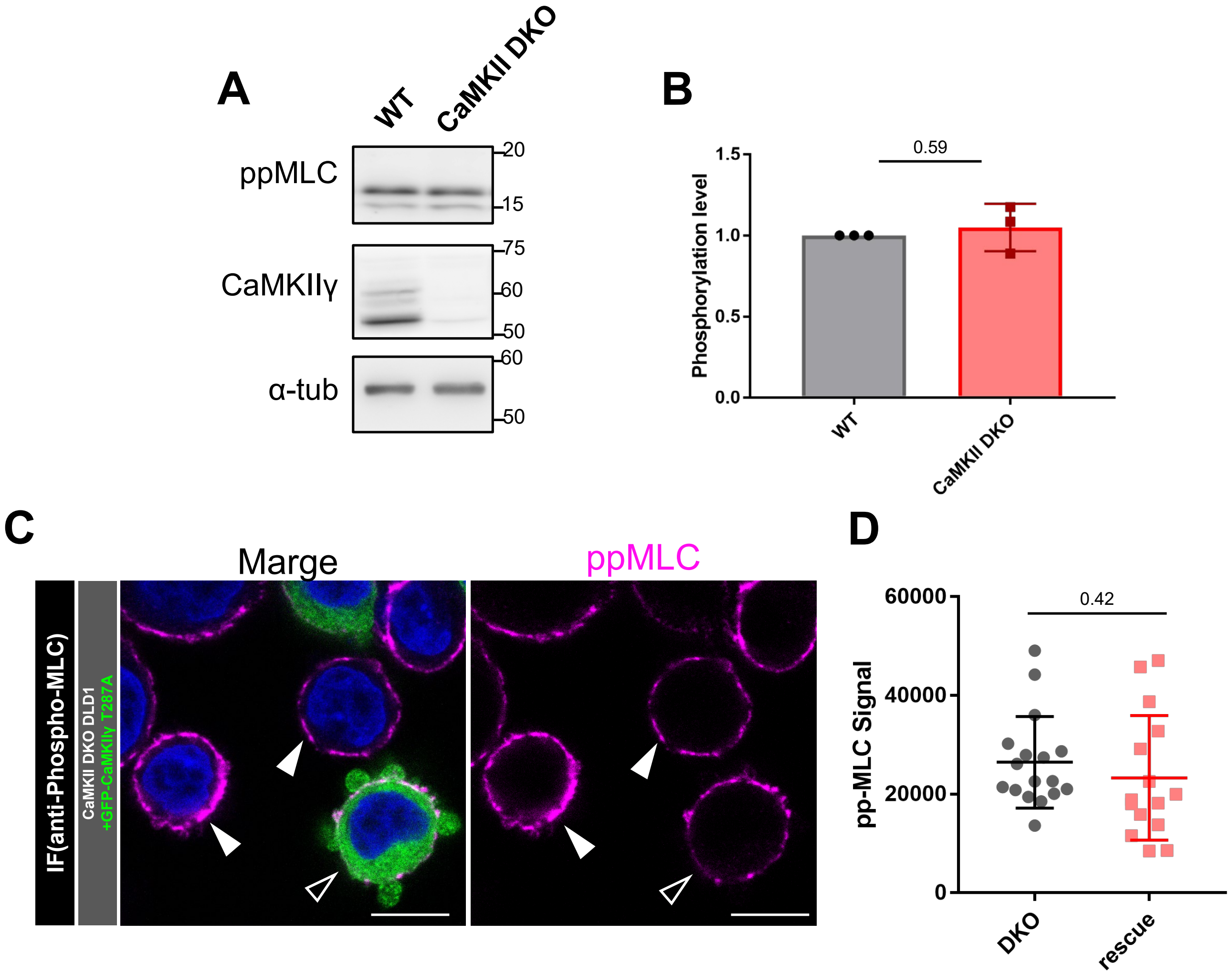

Appendix Figure S3

Contractility of Actomyosin Cortex Remains Unaltered in CaMKII DKO Cells

(A) Western blotting revealed comparable levels of phosphorylated myosin light chain (pp-MLC) (an indicator of actomyosin contractility) in WT and CaMKII DKO cells. α-tubulin served as a loading control, and the absence of CaMKIIγ in DKO cells was verified by an anti-CaMKIIγ antibody.

(B) The ratio of pp-MLC signal intensity, normalized to α-tubulin, in CaMKII DKO cells compared to WT cells (set to 1). Statistical analysis revealed no significant difference in myosin activation between these cell lines ; exact *p* value is $p = 0.5926$.

(C) CaMKII DKO cells—some expressing GFP-hCaMKIIγ T287A—were immunostained with anti-pp-MLC antibody. White arrowheads identify CaMKII DKO cells, while black arrowheads denote “rescue” cells expressing GFP-hCaMKIIγ T287A.

(D) A line-scan analysis of the cortical actin region in the cell body was performed to quantify pp-MLC signal intensity in CaMKII DKO cells (N=17) versus rescue cells (N=15). These results collectively indicate that the contractile force of the actin cortex within the cell body is not substantially altered by CaMKII knockout. Results are shown as mean of four biological replicates ± SD and the *p* values of Student’s *t*-test are indicated; exact *p* value is $p = 0.4213$.

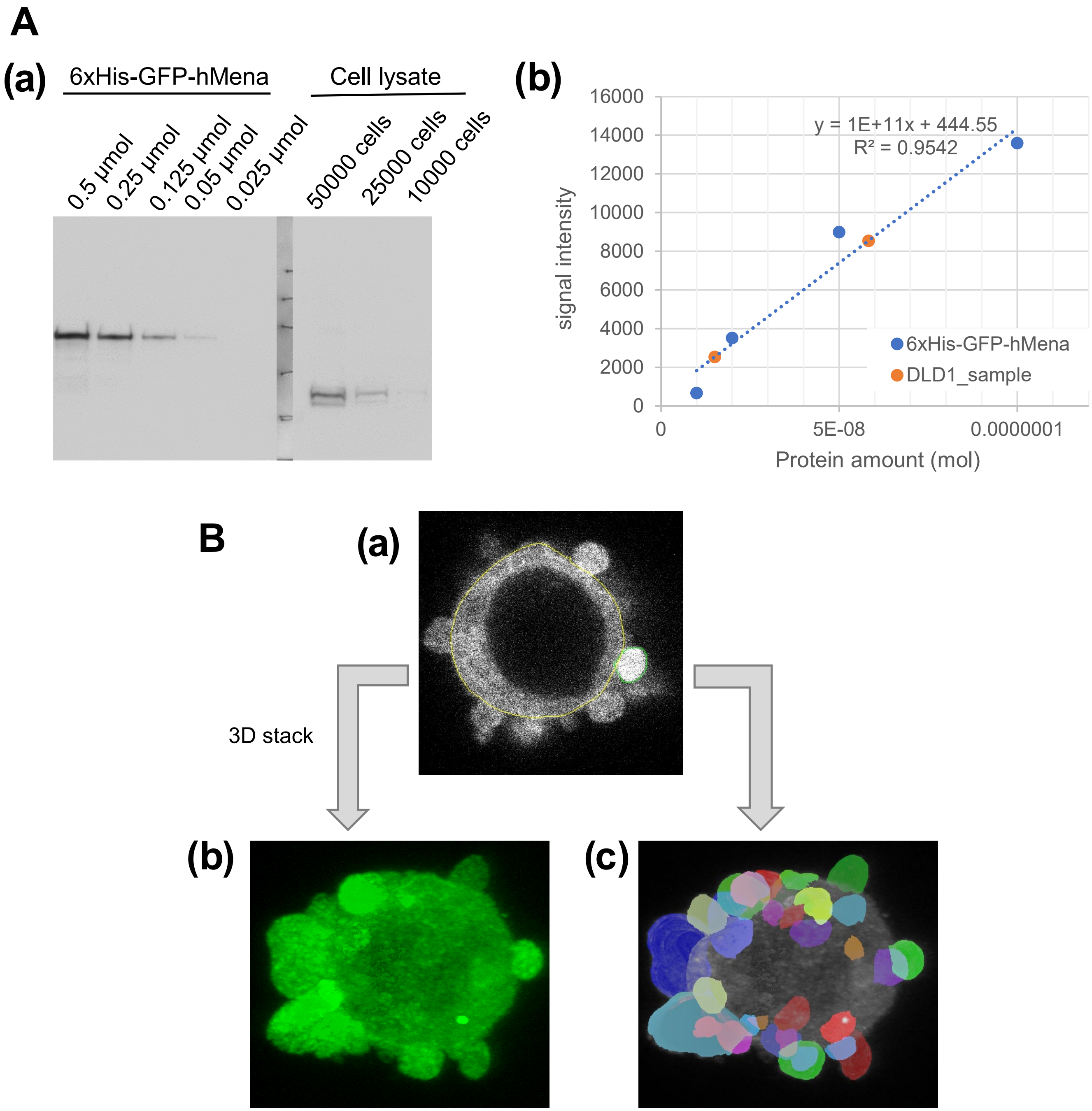

Appendix Figure S4

Estimation of Protein Concentration in Blebs versus the Cell Body

(A) To determine the amount of a given protein per cell, we purified and quantified 6×His-GFP-tagged versions of the protein of interest in E. coli, then compared these standards to serially diluted total lysates of DLD1 cells. Taking Mena as an example, we performed Western blotting with an anti-Mena antibody on both the purified protein standard and carefully normalized DLD1 lysates (a). From these results, we calculated the per-cell expression level of Mena (b). This entire procedure was repeated three times, yielding an estimate of the total Mena protein per cell.

(B) For three-dimensional analysis of blebbing cells, we measured the intracellular distribution of CaMKII as follows. CaMKII DKO DLD1 cells expressing GFP-hCaMKIIγ T287A were fixed, stained by immunofluorescence, and imaged in 3D at 0.2-μm slice intervals. Regions of interest (ROIs)—nucleus, cell body, and individual blebs—were manually defined (a). For each ROI, the average fluorescence intensity and projected area were used to compute the fluorescence density and volume, respectively (b). We defined the cell-body cytoplasm average fluorescence intensity as 1, and calculated the relative intensity in each bleb. Using this relative intensity, the bleb volume, and the total CaMKII expression level per cell (obtained from the purified standards), we derived the estimated CaMKII concentration in each region (c). Proteins that accumulate in expanding blebs in a CaMKII-dependent manner were assumed to follow a distribution similar to that of CaMKII; we thus computed their bleb and cell-body concentrations and determined the concentration differences between these compartments. Further assuming that each protein contributes equally to the osmotic pressure, we defined these proteins collectively as "osmotic pressure–driving proteins," and used their cumulative concentration differences to validate our mathematical model.

Appendix TableS1-1

	Amount of osmolyte per single cell (10^{-18}mol)
CaMKII	1.69 ± 0.36
Mena	0.96 ± 0.12
VASP	1.23 ± 0.17
ERK1	3.03 ± 3.24
total	6.15 ± 2.74

Appendix Table S1-2

	CaMKII	Total osmolyte
Cell volume without nucleus (μm³)	1363.94 ± 278.30	
Estimated osmolyte concentration in cell body (μM)	1.20 ± 0.18	4.35 ± 0.66
Estimated osmolyte concentration in expanding bleb (μM)	2.19 ± 0.51	7.94 ± 1.84
Estimated difference in concentration of endogenous osmolyte (μM)	0.99 ± 0.46	3.59 ± 1.65

Appendix Table S1-3

	WT	CaMKII DKO
Cell volume without nucleus (μm³)	1400.32 ± 638.62	1514.65 ± 398.84
Average bleb volume of top 10 % of each cell (μm³)	50.84 ± 23.66	13.04 ± 7.36
Theoretical bleb radius (μm)	2.78	1.78

Appendix Table S1

Parameters Used to Define Bleb Characteristics for Mathematical Model Construction

(1-1) Per-Cell Protein Abundance (N=3): Measured levels of CaMKII and the proteins that accumulate in expanding blebs in a CaMKII-dependent manner, determined on a per-cell basis.

(1-2) 3D Cell Volume and Protein Concentrations (N=3): Volumes and local protein concentrations calculated from three-dimensional reconstructions of CaMKII DKO DLD1 cells expressing GFP-hCaMKII T287A.

(1-3) Comparison of WT and CaMKII DKO DLD1 Cells: Overall cell volume and the average volume of the largest 10% of blebs. Assuming a hemispherical geometry for blebs, the radius was estimated based on the average bleb volume.
