# Supplementary figures and images for: CaMKII nucleates an osmotic protein supercomplex to induce cellular bleb expansion
Source: EMBO J. 2026 Feb 3;45(8):2433–55. doi: 10.1038/s44318-026-00703-5 (PMC13083957; doi:10.1038/s44318-026-00703-5)

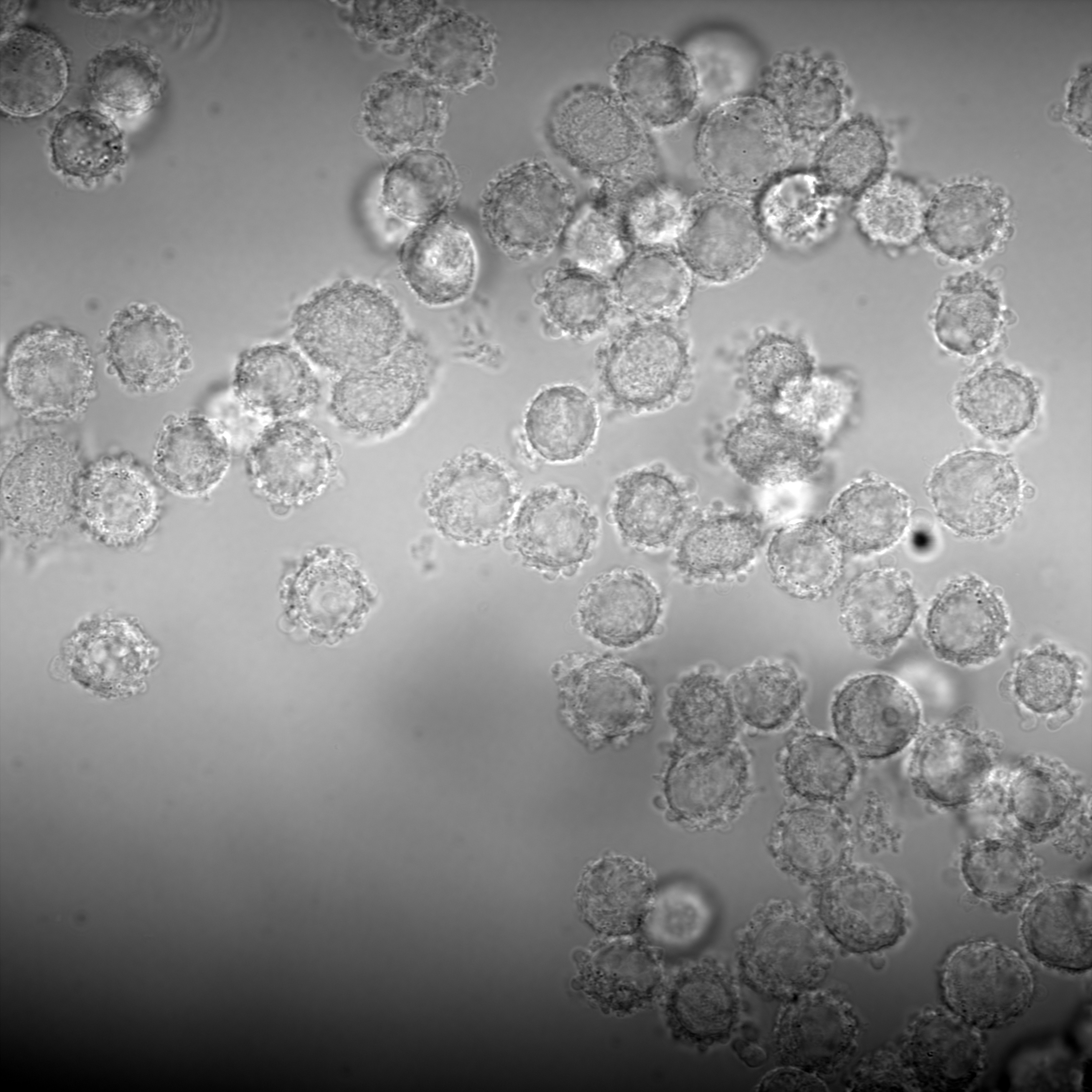

Supplement: Supplementary file 6 — Source data Fig. 1 [file 44318_2026_703_MOESM6_ESM.zip › Fig1/1A/1A_AncoA4.tif]

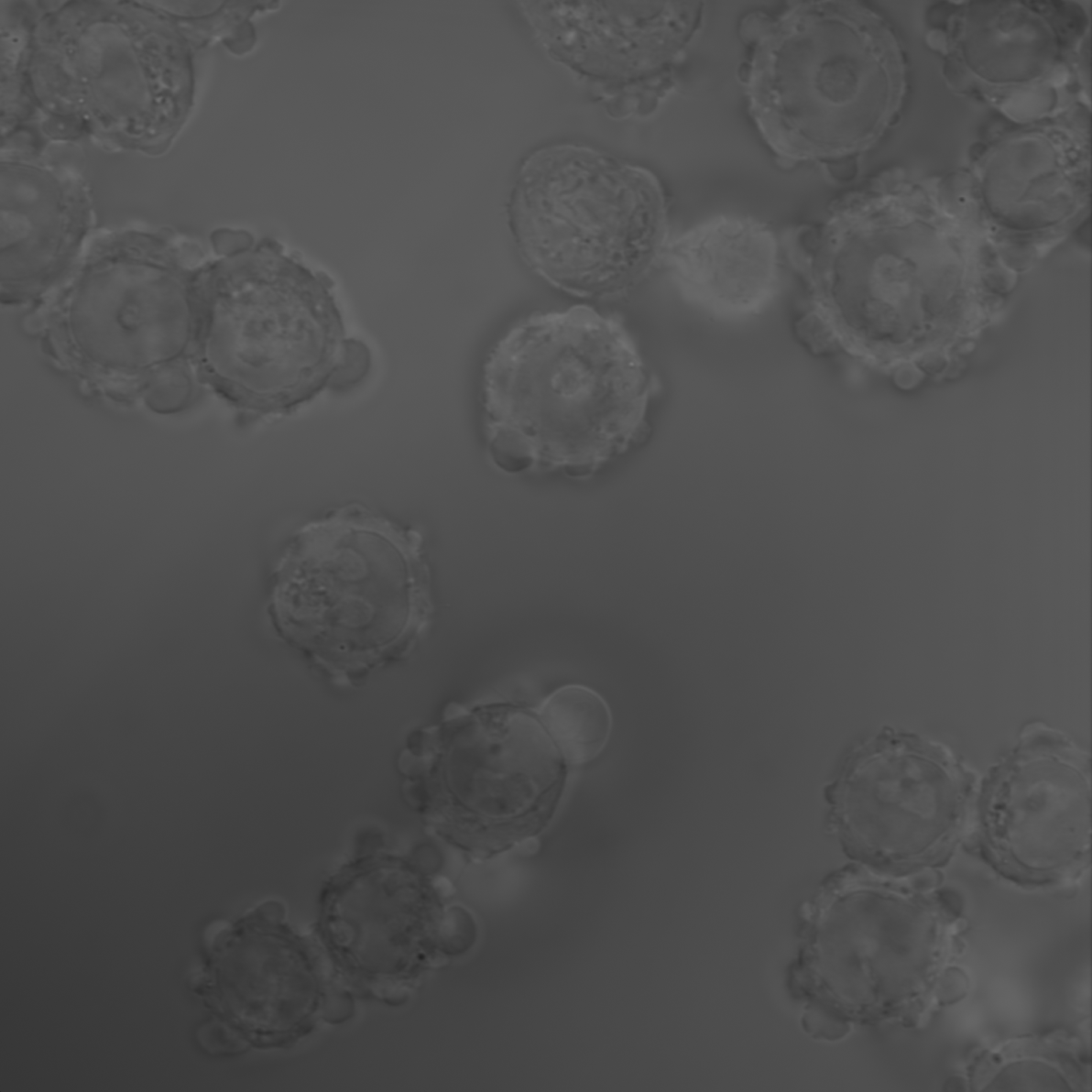

Supplement: Supplementary file 6 — Source data Fig. 1 [file 44318_2026_703_MOESM6_ESM.zip › Fig1/1A/1A_DMSO.tif]

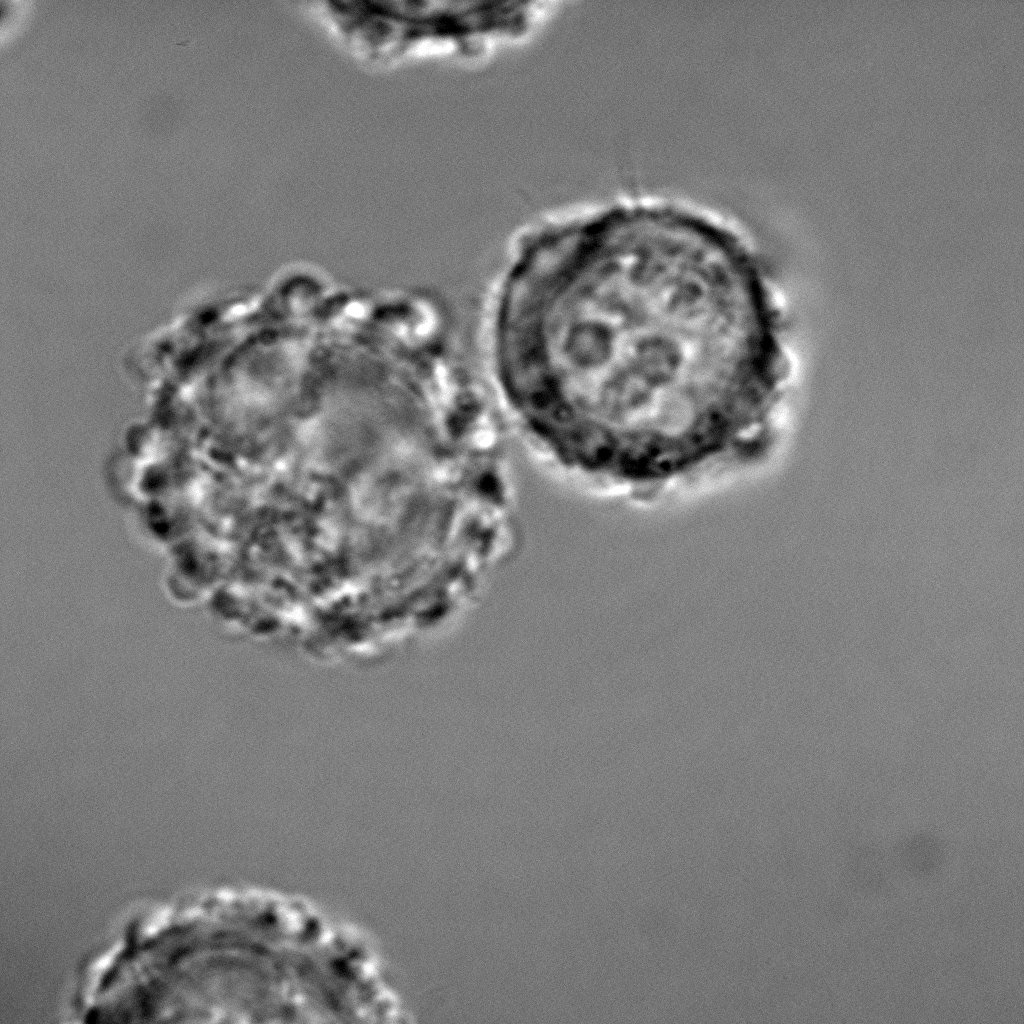

Supplement: Supplementary file 6 — Source data Fig. 1 [file 44318_2026_703_MOESM6_ESM.zip › Fig1/1A/1A_EDTA.tif]

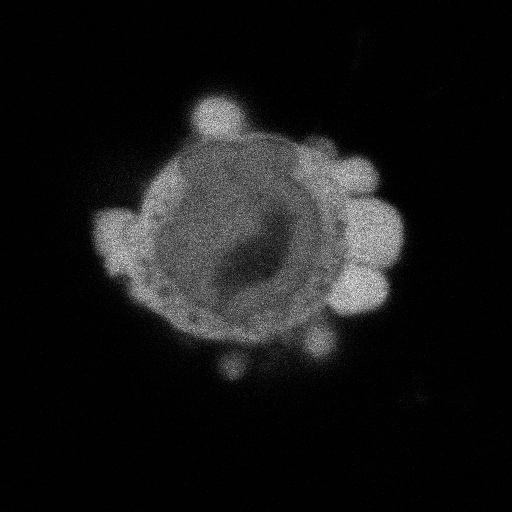

Supplement: Supplementary file 6 — Source data Fig. 1 [file 44318_2026_703_MOESM6_ESM.zip › Fig1/1C/1C_GCaMP_timelaps.tif]

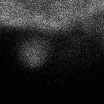

Supplement: Supplementary file 6 — Source data Fig. 1 [file 44318_2026_703_MOESM6_ESM.zip › Fig1/1C/1C_GCaMP_timelaps_cropped.tif]

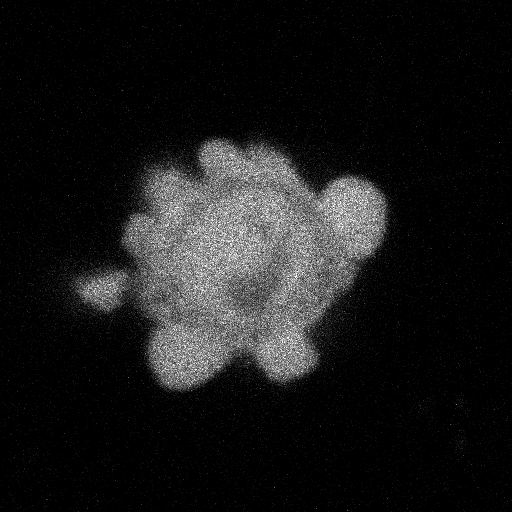

Supplement: Supplementary file 6 — Source data Fig. 1 [file 44318_2026_703_MOESM6_ESM.zip › Fig1/1D/1D_GCaMP-RFP.tif]

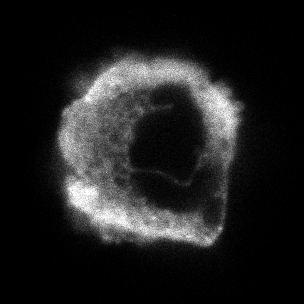

Supplement: Supplementary file 6 — Source data Fig. 1 [file 44318_2026_703_MOESM6_ESM.zip › Fig1/1G/1G_GFP-CaMKIIγ_Lifeact_timelaps.tif]

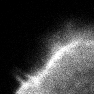

Supplement: Supplementary file 6 — Source data Fig. 1 [file 44318_2026_703_MOESM6_ESM.zip › Fig1/1G/1G_GFP-CaMKIIγ_Lifeact_timelaps_crop.tif]

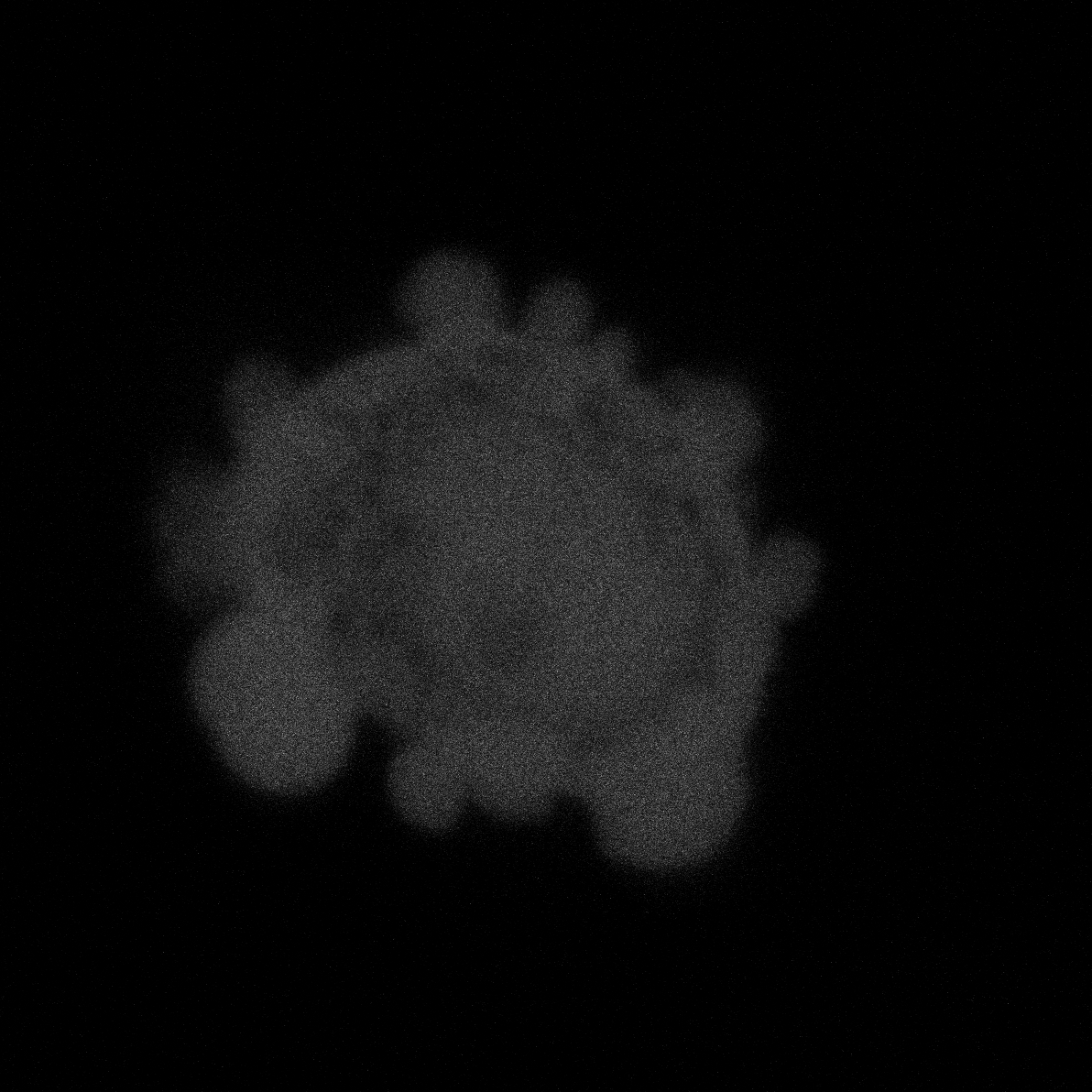

Supplement: Supplementary file 6 — Source data Fig. 1 [file 44318_2026_703_MOESM6_ESM.zip › Fig1/1H/1H_GFP-CaMKIIγ-RFP.tif]

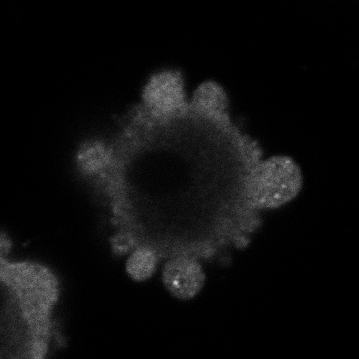

Supplement: Supplementary file 6 — Source data Fig. 1 [file 44318_2026_703_MOESM6_ESM.zip › Fig1/1J/1J__IF-CaMKIIγ.tif]

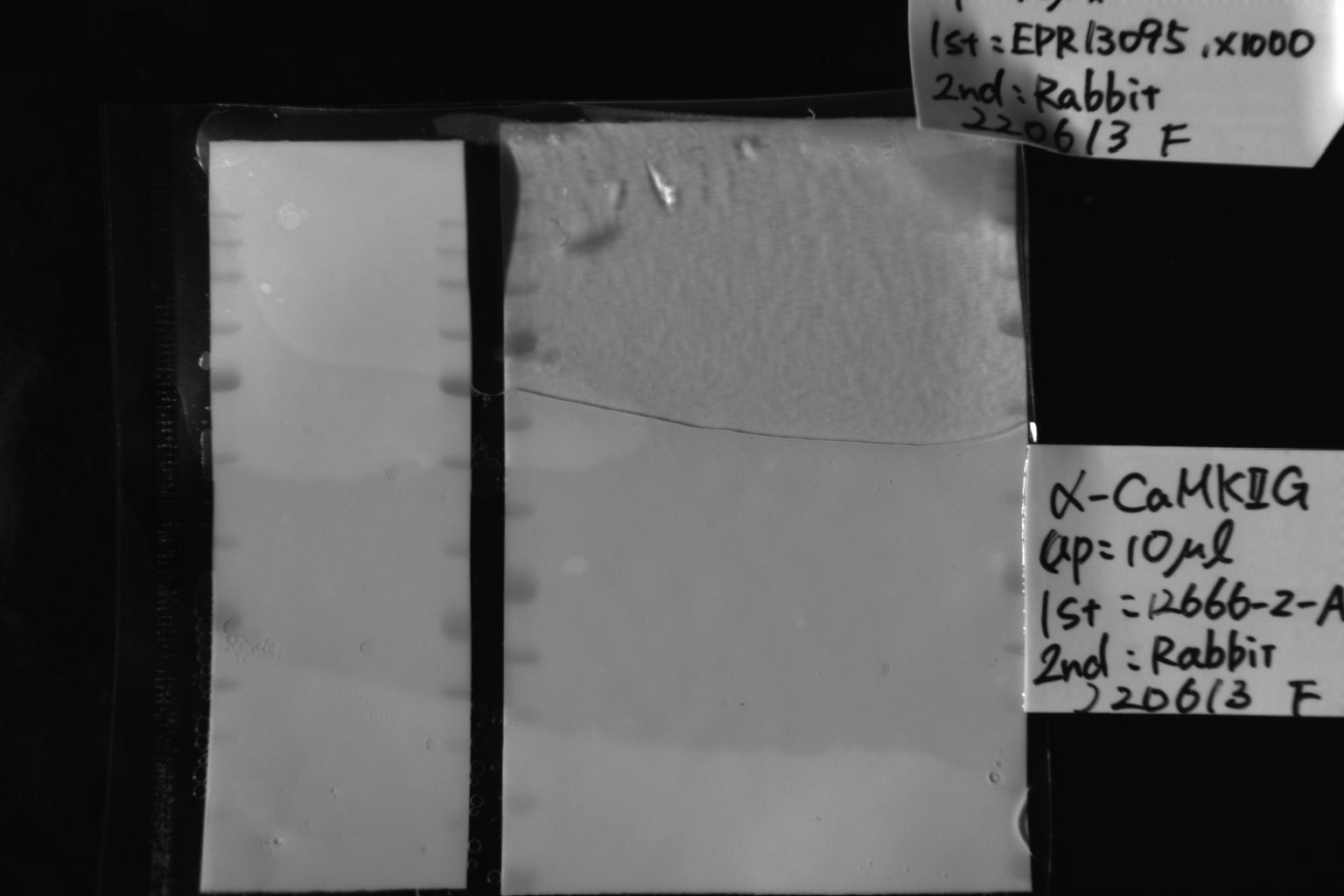

Supplement: Supplementary file 7 — Source data Fig. 2 [file 44318_2026_703_MOESM7_ESM.zip › Fig2/2A/2A_uncropped WB/2A-1'_WB CaMKIIγ_marker.tif]

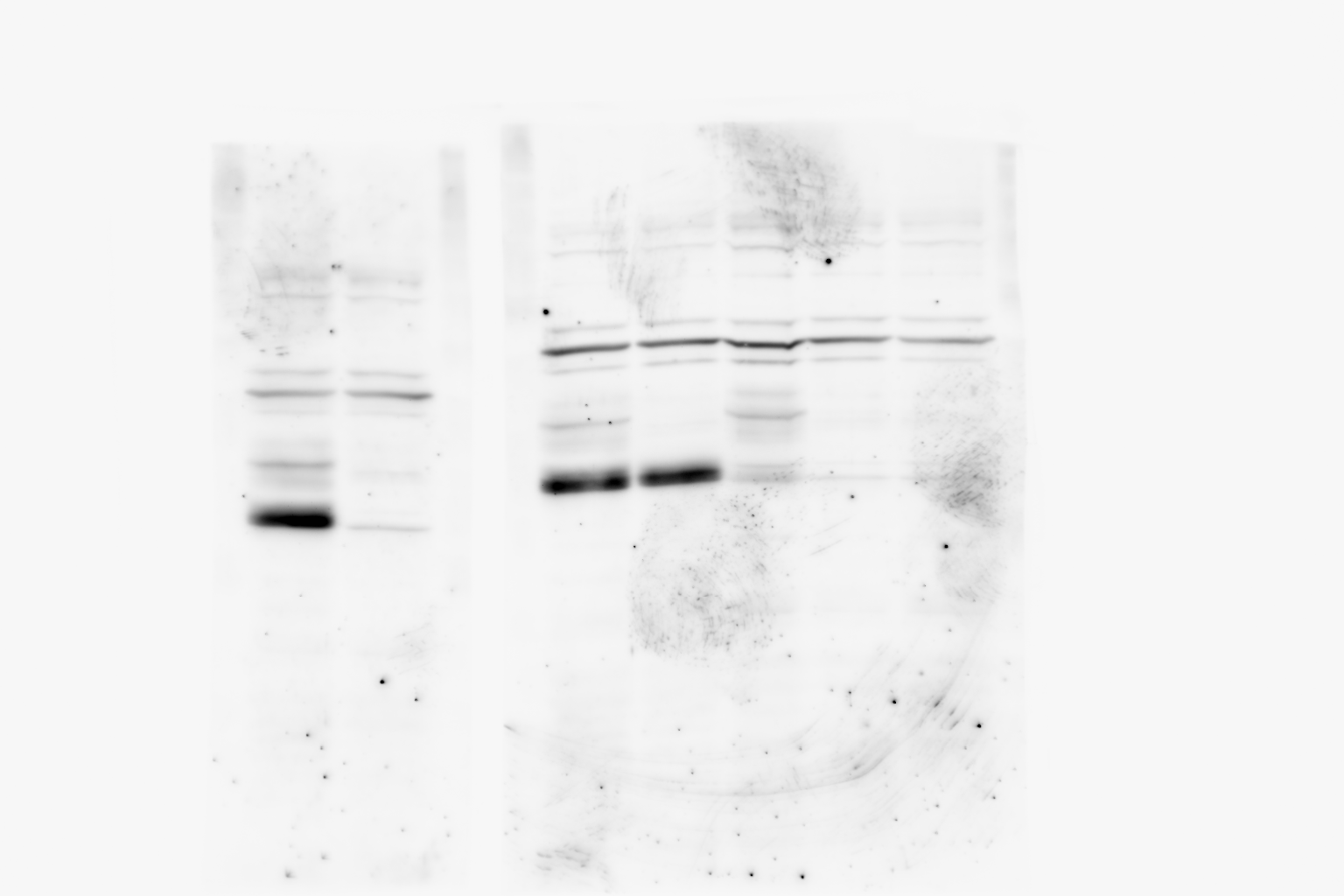

Supplement: Supplementary file 7 — Source data Fig. 2 [file 44318_2026_703_MOESM7_ESM.zip › Fig2/2A/2A_uncropped WB/2A-1_WB CaMKIIγ.tif]

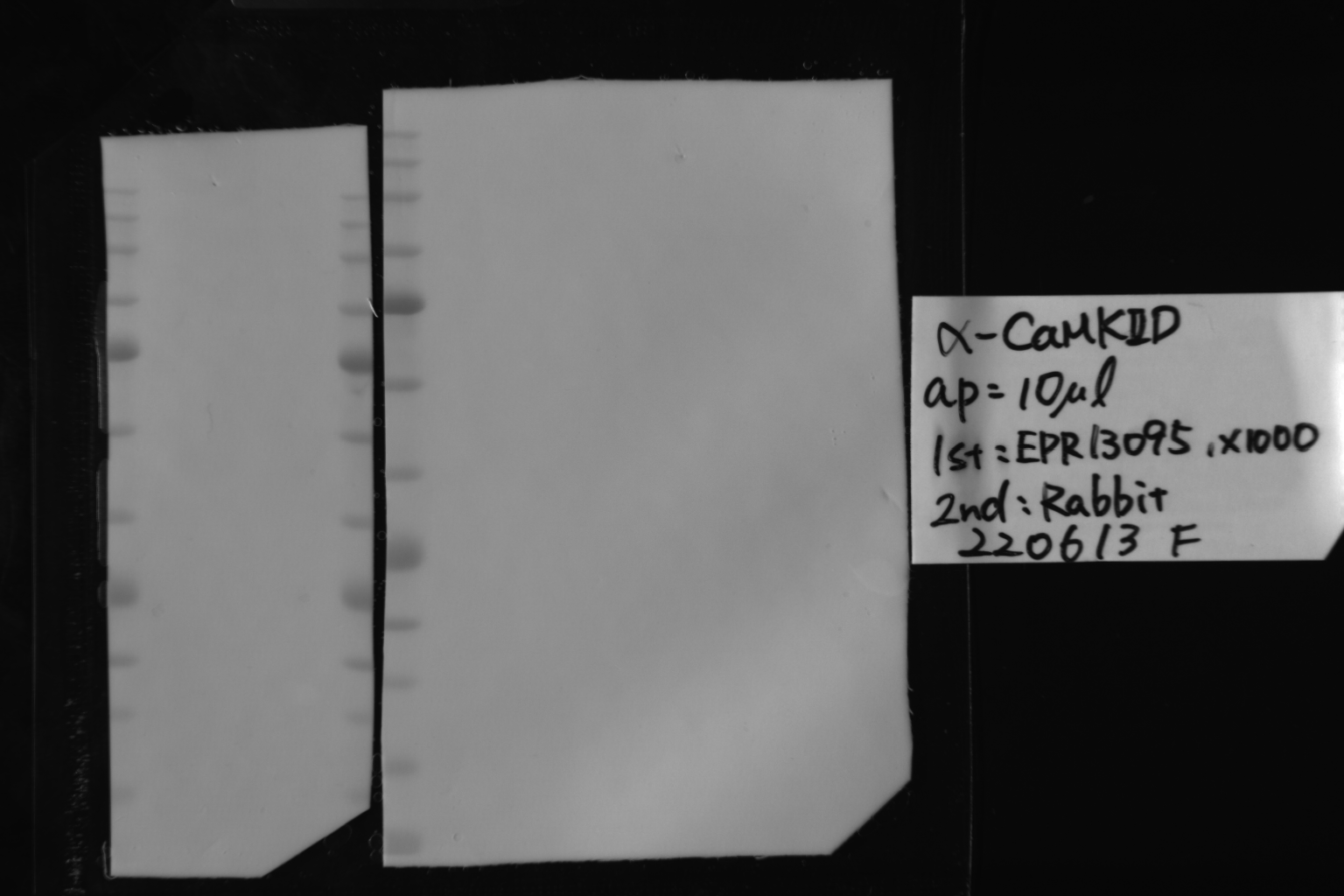

Supplement: Supplementary file 7 — Source data Fig. 2 [file 44318_2026_703_MOESM7_ESM.zip › Fig2/2A/2A_uncropped WB/2A-2'_WB CaMKIIδ_marker.tif]

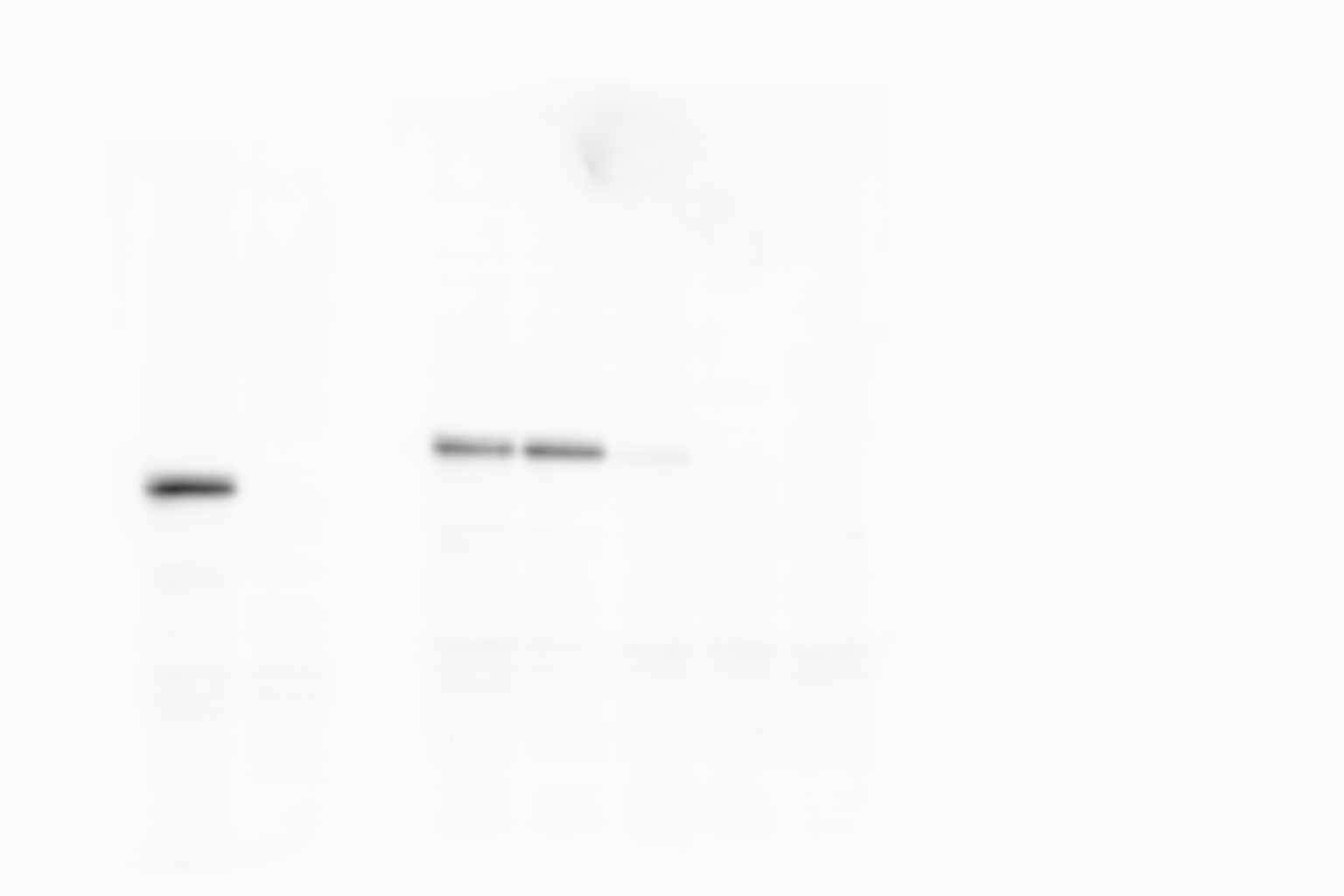

Supplement: Supplementary file 7 — Source data Fig. 2 [file 44318_2026_703_MOESM7_ESM.zip › Fig2/2A/2A_uncropped WB/2A-2_WB CaMKIIδ.tif]

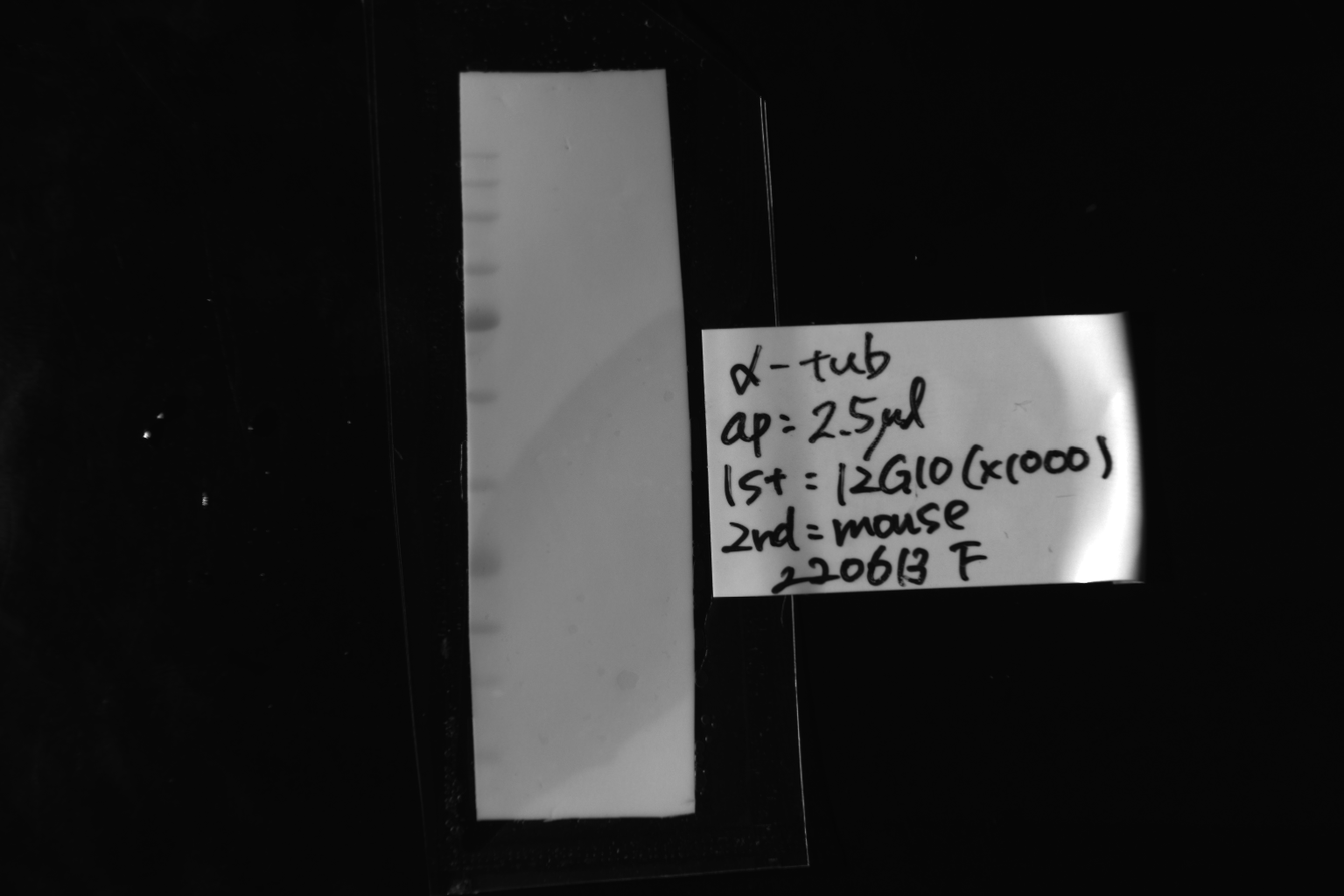

Supplement: Supplementary file 7 — Source data Fig. 2 [file 44318_2026_703_MOESM7_ESM.zip › Fig2/2A/2A_uncropped WB/2A-3'_WB α-tublin_marker.tif]

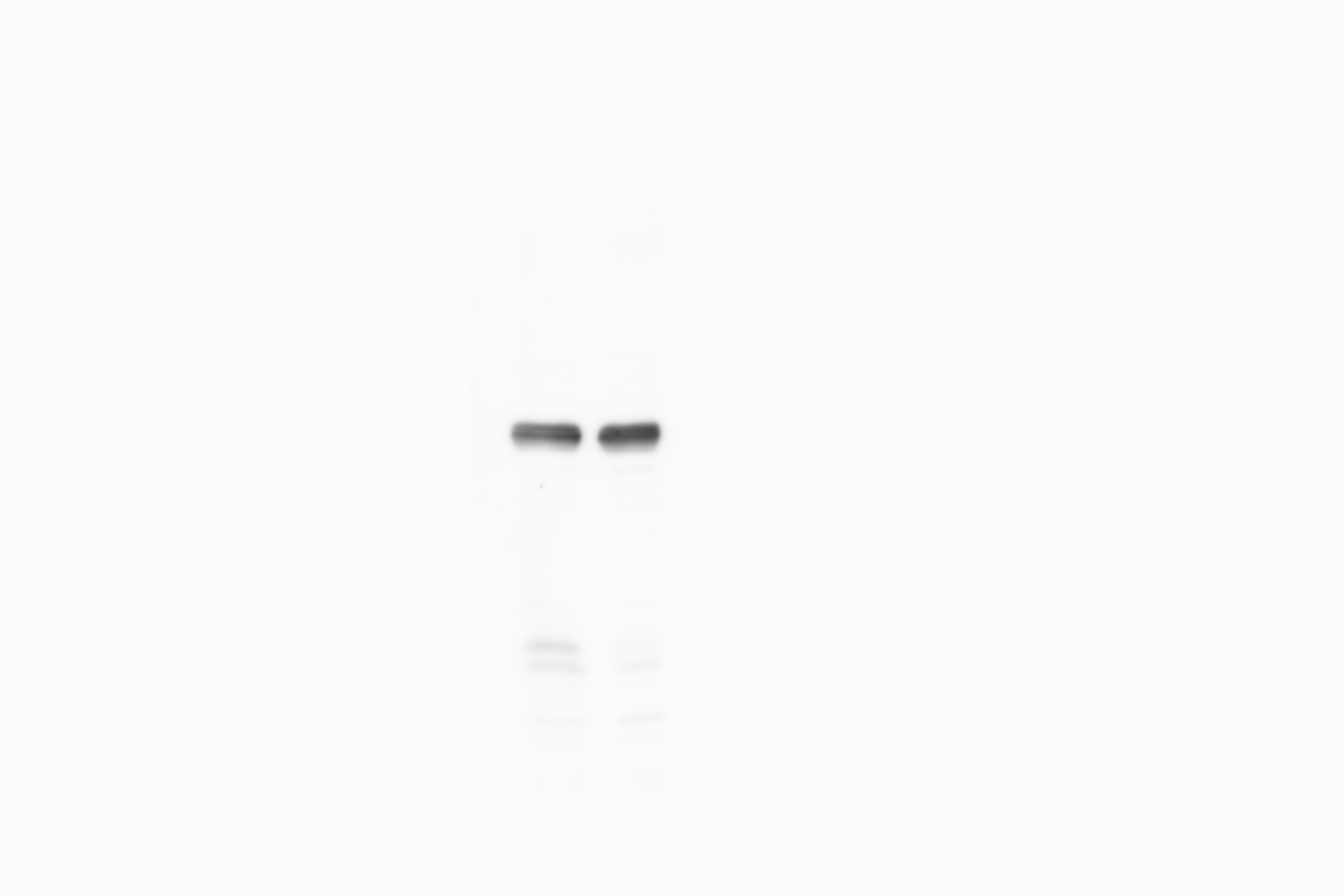

Supplement: Supplementary file 7 — Source data Fig. 2 [file 44318_2026_703_MOESM7_ESM.zip › Fig2/2A/2A_uncropped WB/2A-3_WB α-tublin.tif]

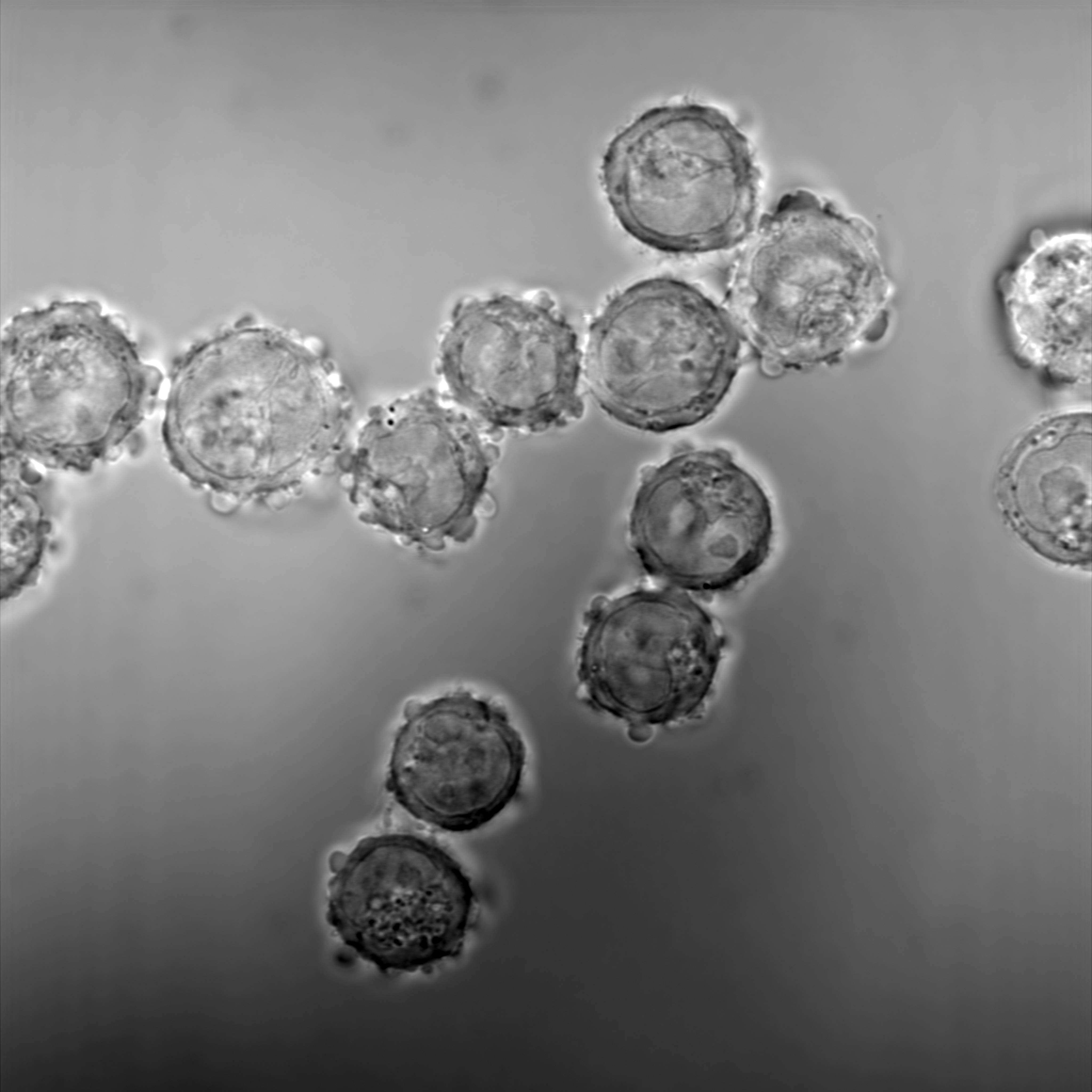

Supplement: Supplementary file 7 — Source data Fig. 2 [file 44318_2026_703_MOESM7_ESM.zip › Fig2/2B/2B_DKO.tif]

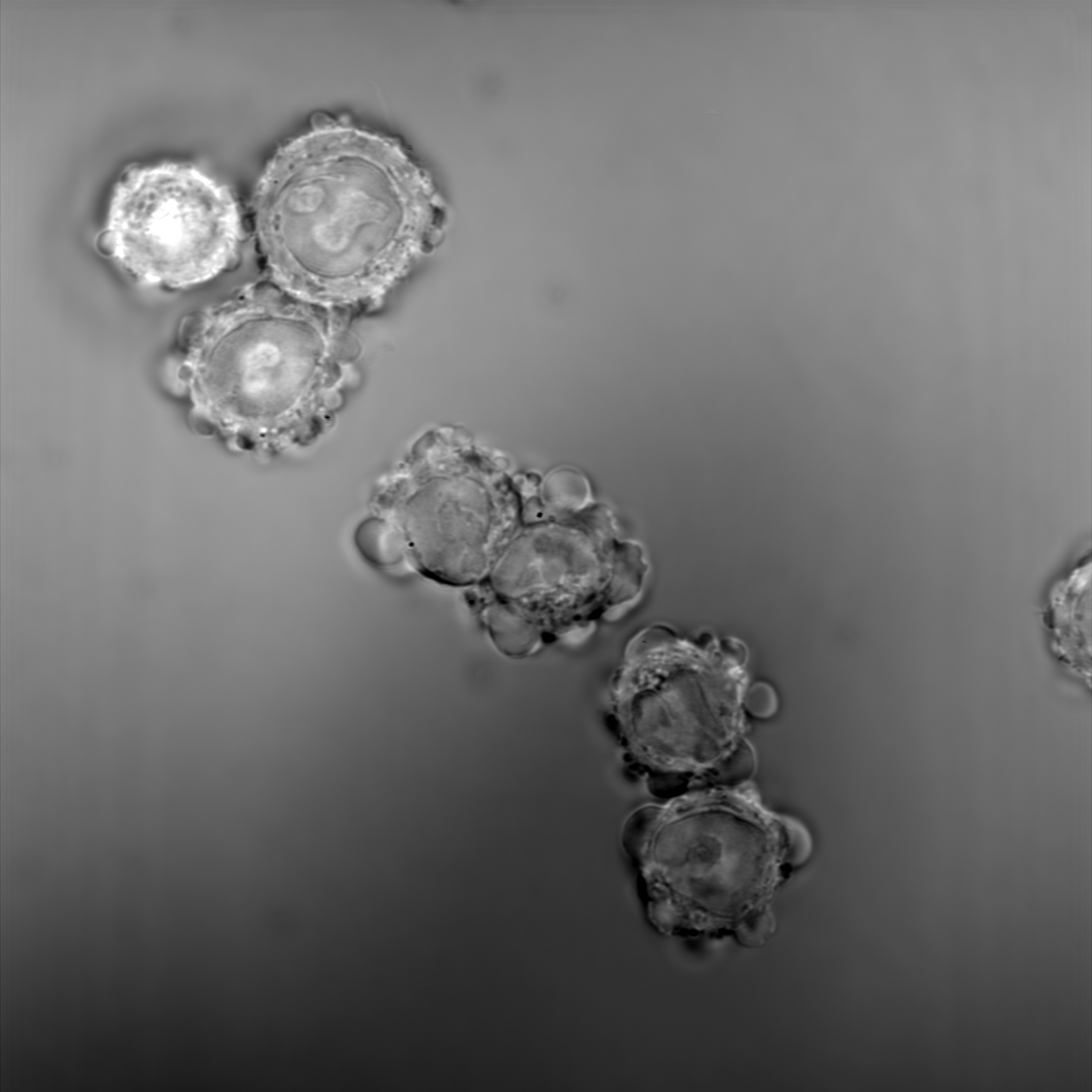

Supplement: Supplementary file 7 — Source data Fig. 2 [file 44318_2026_703_MOESM7_ESM.zip › Fig2/2B/2B_WT DLD1_.tif]

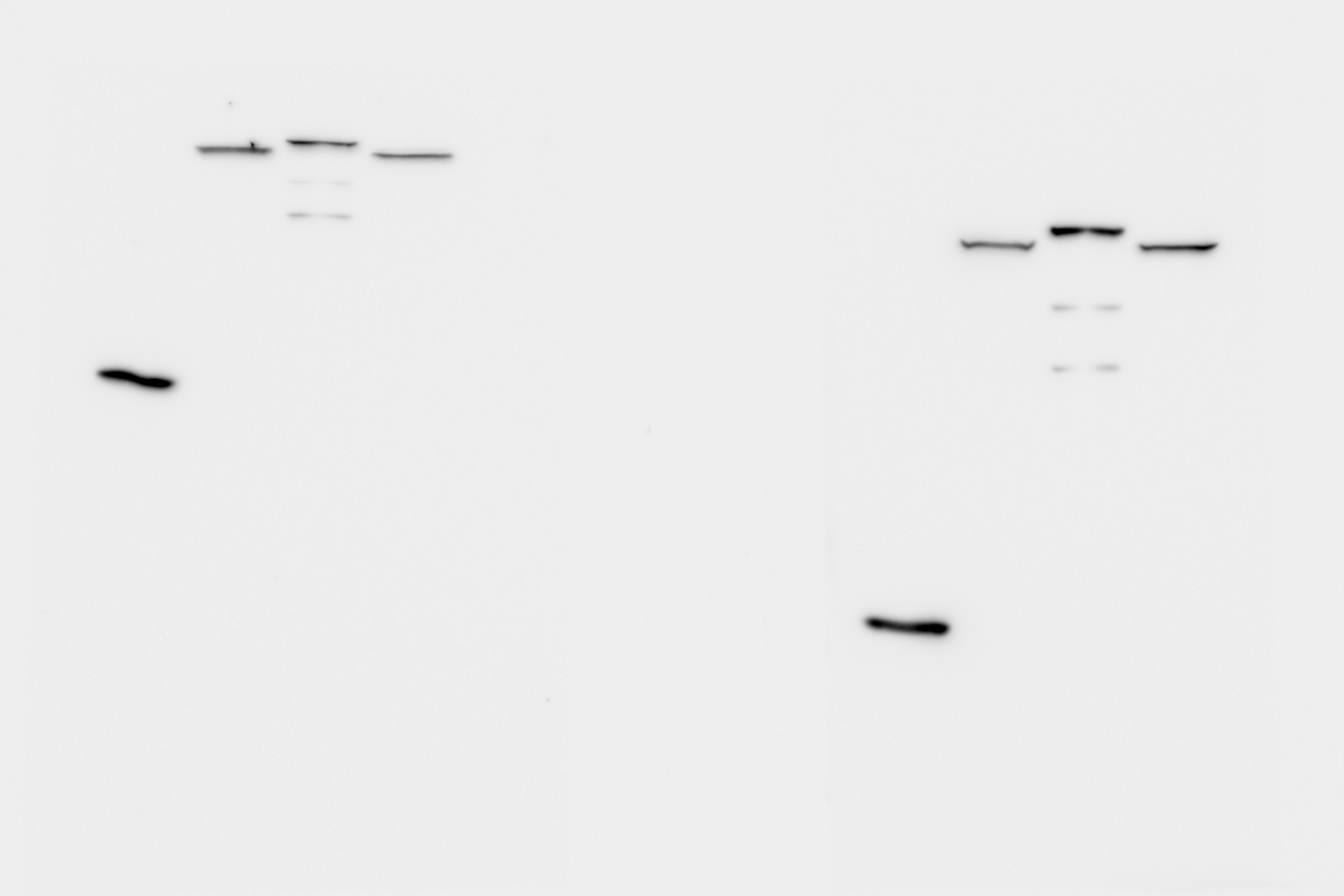

Supplement: Supplementary file 8 — Source data Fig. 3 [file 44318_2026_703_MOESM8_ESM.zip › Fig3/3B/uncropped WB/3B_WB GFP.tif]

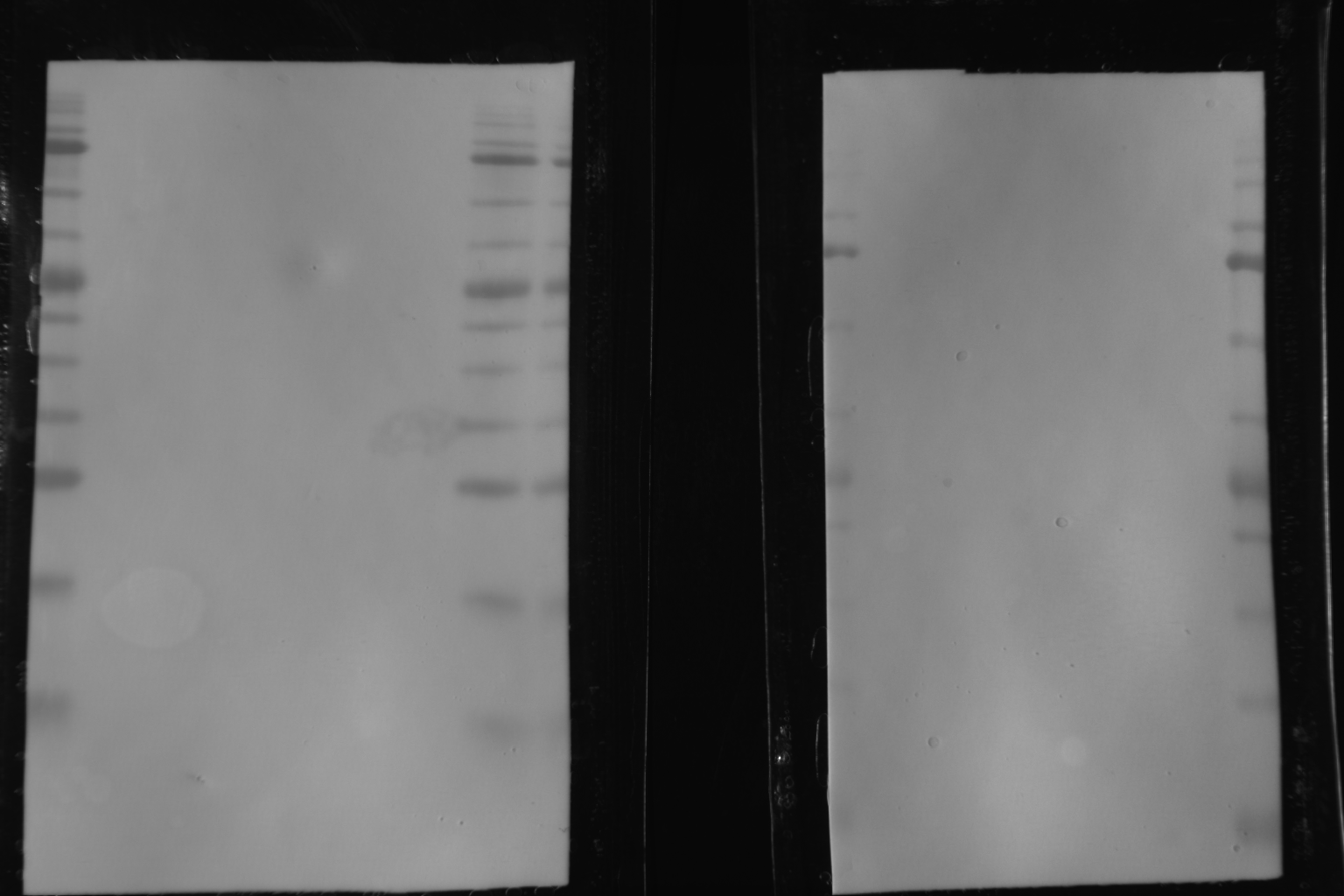

Supplement: Supplementary file 8 — Source data Fig. 3 [file 44318_2026_703_MOESM8_ESM.zip › Fig3/3B/uncropped WB/3B_WB GFP_marker.tif]

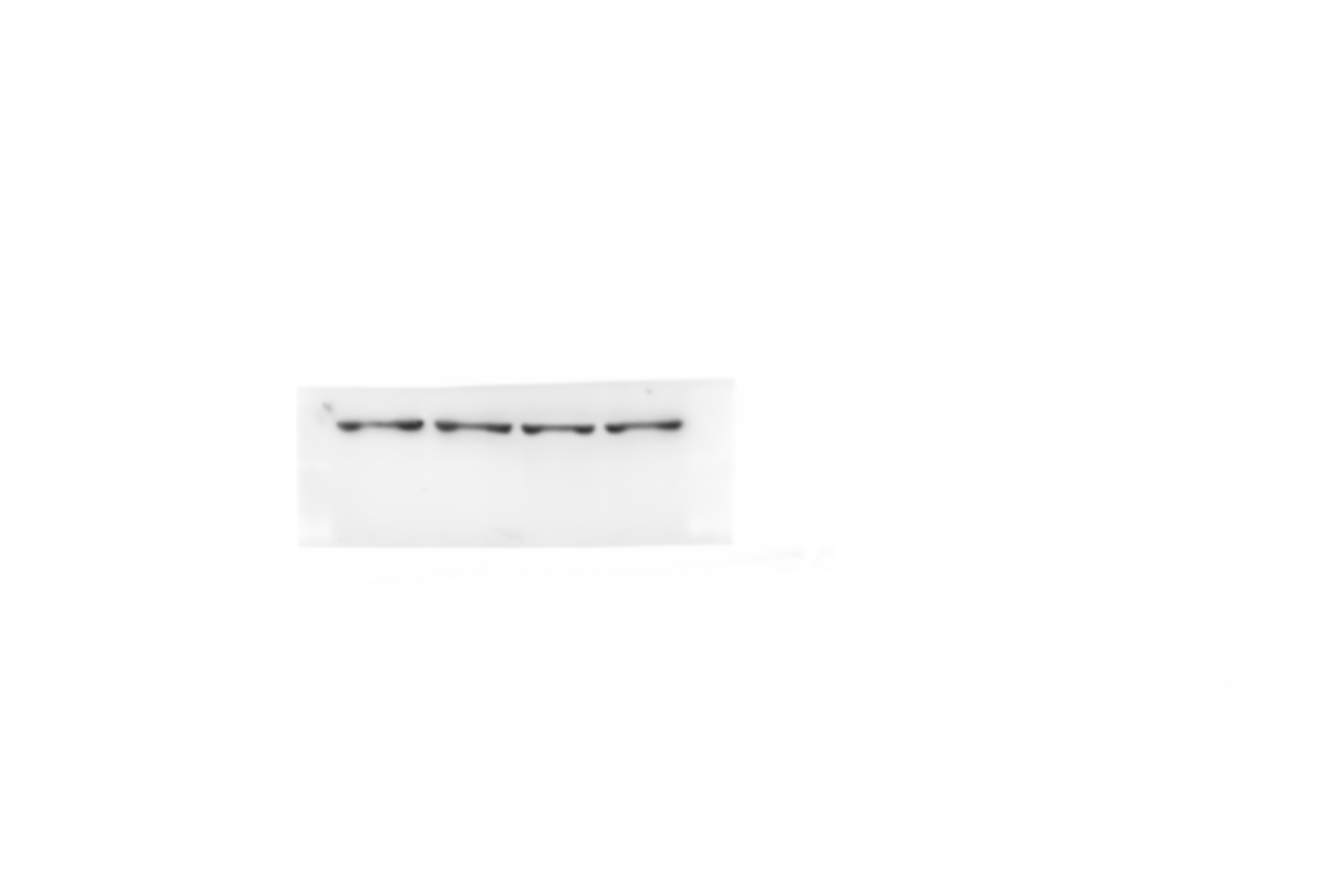

Supplement: Supplementary file 8 — Source data Fig. 3 [file 44318_2026_703_MOESM8_ESM.zip › Fig3/3B/uncropped WB/3B_WB α-tubulin.tif]

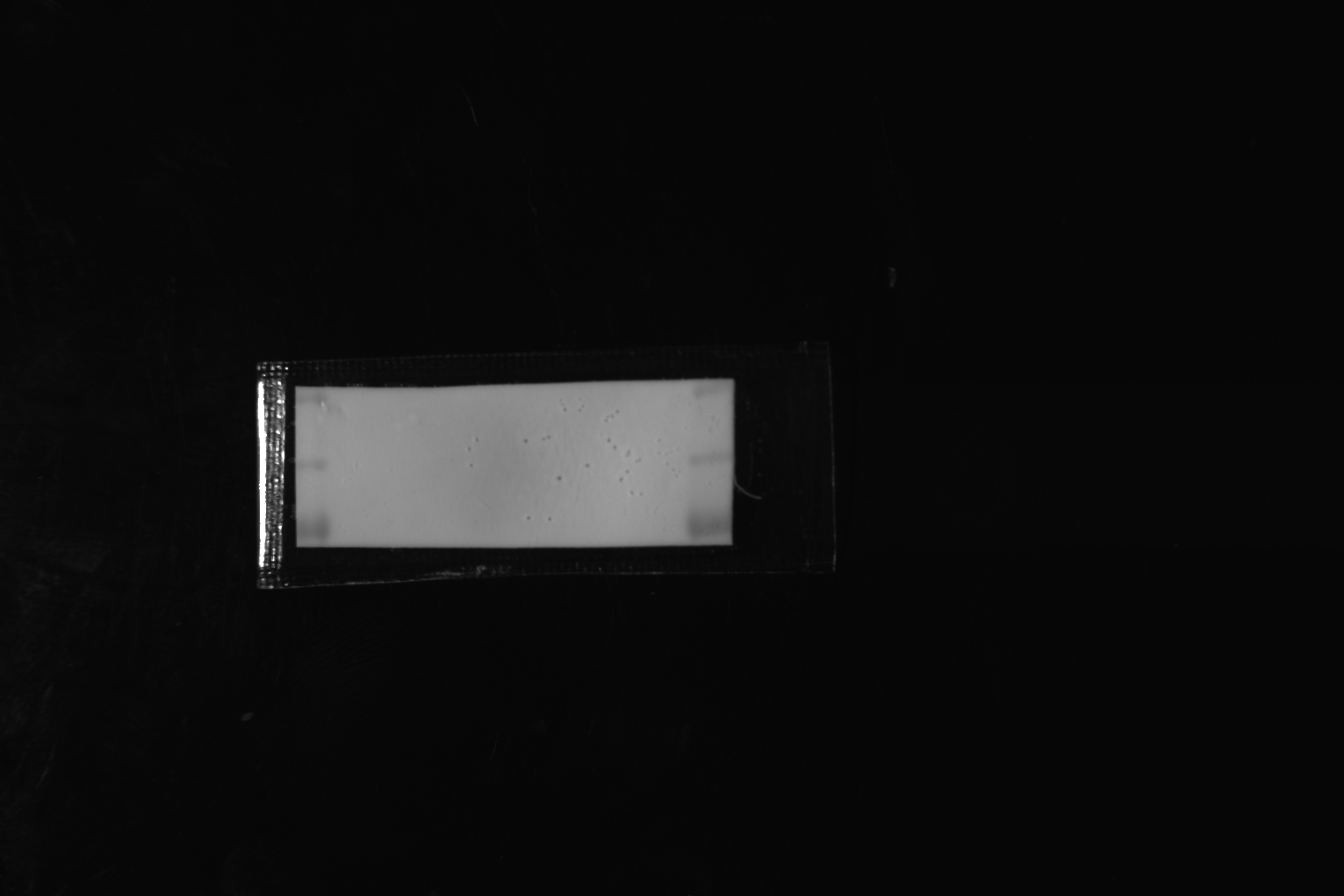

Supplement: Supplementary file 8 — Source data Fig. 3 [file 44318_2026_703_MOESM8_ESM.zip › Fig3/3B/uncropped WB/3B_WB α-tubulin_marker.tif]

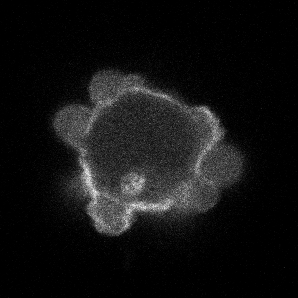

Supplement: Supplementary file 8 — Source data Fig. 3 [file 44318_2026_703_MOESM8_ESM.zip › Fig3/3D/3D_GFP-CaMKIIγ T287A,K43M_Lifeact.tif]

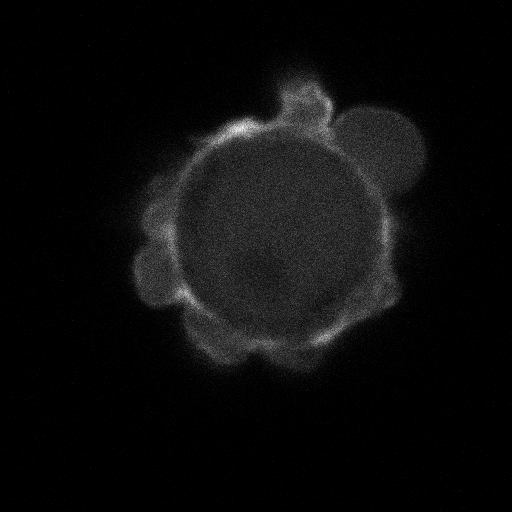

Supplement: Supplementary file 8 — Source data Fig. 3 [file 44318_2026_703_MOESM8_ESM.zip › Fig3/3D/3D_GFP-CaMKIIγ T287A_Lifeact.tif]

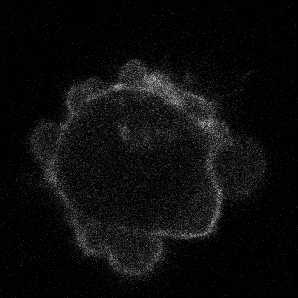

Supplement: Supplementary file 8 — Source data Fig. 3 [file 44318_2026_703_MOESM8_ESM.zip › Fig3/3D/3D_GFP-CaMKIIγ_Lifeact.tif]

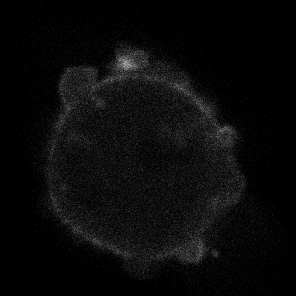

Supplement: Supplementary file 8 — Source data Fig. 3 [file 44318_2026_703_MOESM8_ESM.zip › Fig3/3D/3D_GFP_Lifeact.tif]

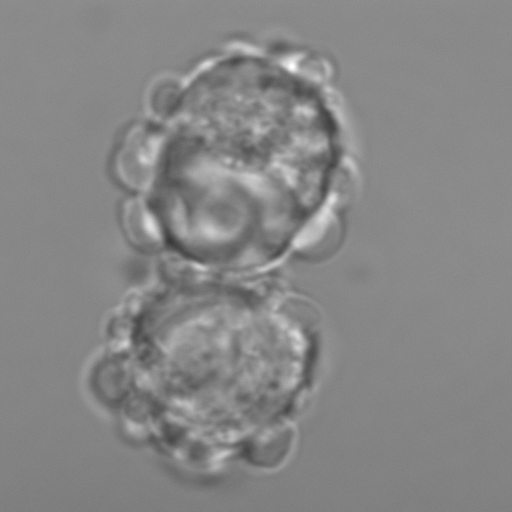

Supplement: Supplementary file 9 — Source data Fig. 4 [file 44318_2026_703_MOESM9_ESM.zip › Fig4/4A/4A_Control.tif]

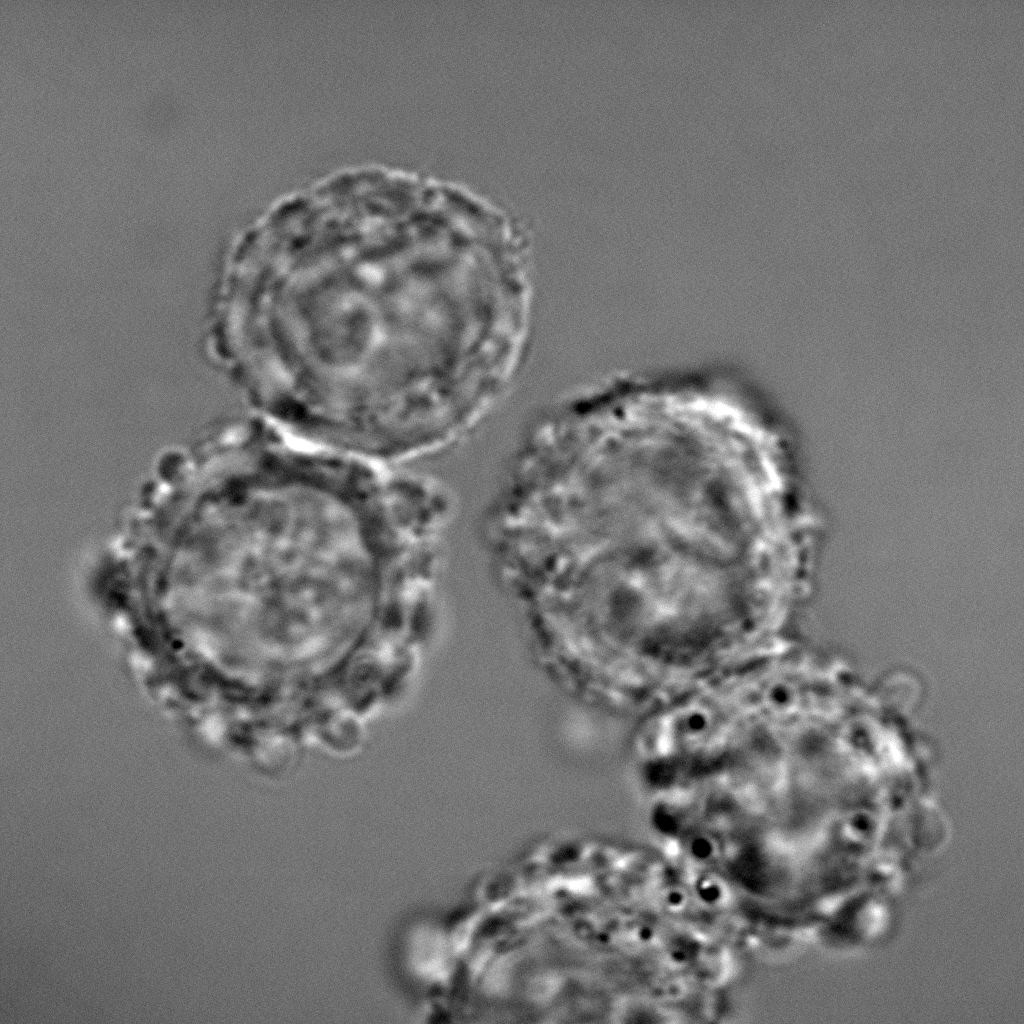

Supplement: Supplementary file 9 — Source data Fig. 4 [file 44318_2026_703_MOESM9_ESM.zip › Fig4/4A/4A_EDTA.tif]

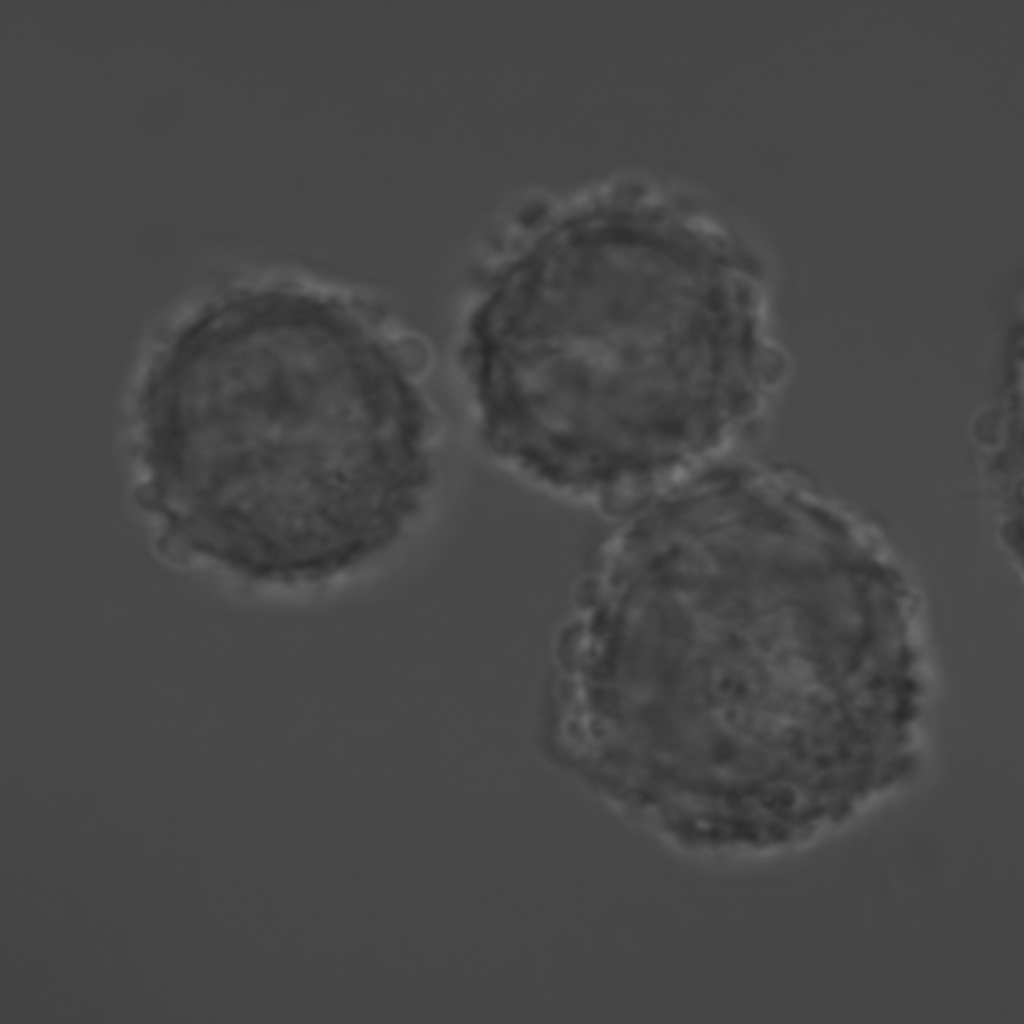

Supplement: Supplementary file 9 — Source data Fig. 4 [file 44318_2026_703_MOESM9_ESM.zip › Fig4/4A/4A_KN93.tif]

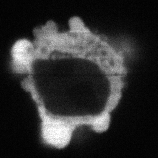

Supplement: Supplementary file 9 — Source data Fig. 4 [file 44318_2026_703_MOESM9_ESM.zip › Fig4/4D/4D_Camui-CR_donor.tif]

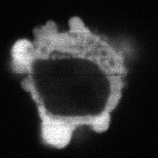

Supplement: Supplementary file 9 — Source data Fig. 4 [file 44318_2026_703_MOESM9_ESM.zip › Fig4/4D/CamuiCR_FRET,donor_Raw.tif]

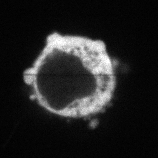

Supplement: Supplementary file 9 — Source data Fig. 4 [file 44318_2026_703_MOESM9_ESM.zip › Fig4/4E/4E_donor/4D_Camui-CR_donor_timelaps.tif]

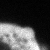

Supplement: Supplementary file 9 — Source data Fig. 4 [file 44318_2026_703_MOESM9_ESM.zip › Fig4/4E/4E_donor/4E_donor_bleb.tif]

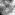

Supplement: Supplementary file 9 — Source data Fig. 4 [file 44318_2026_703_MOESM9_ESM.zip › Fig4/4E/4E_donor/4E_donor_cellbody.tif]

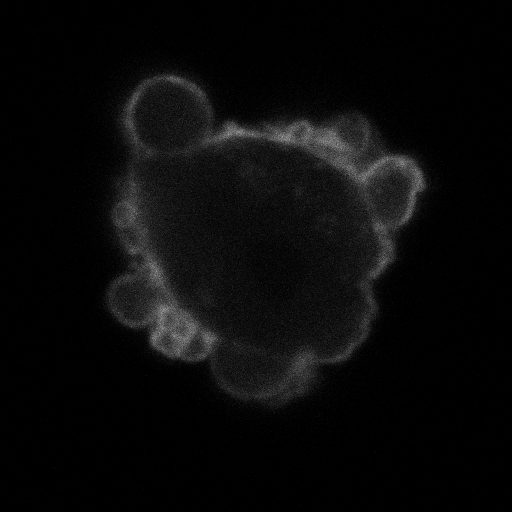

Supplement: Supplementary file 10 — Source data Fig. 5 [file 44318_2026_703_MOESM10_ESM.zip › Fig5/5A/5A_control.tif]

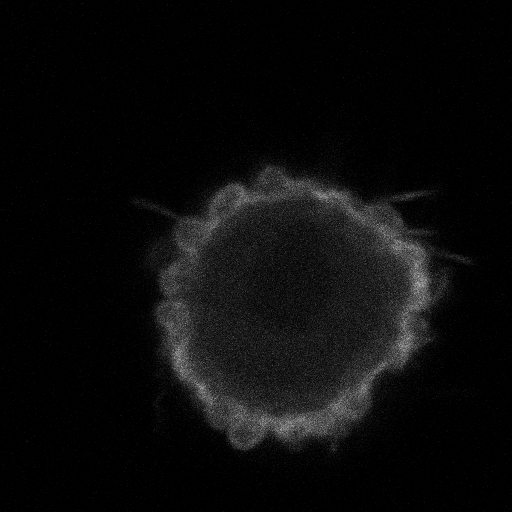

Supplement: Supplementary file 10 — Source data Fig. 5 [file 44318_2026_703_MOESM10_ESM.zip › Fig5/5A/5A_EDTA.tif]

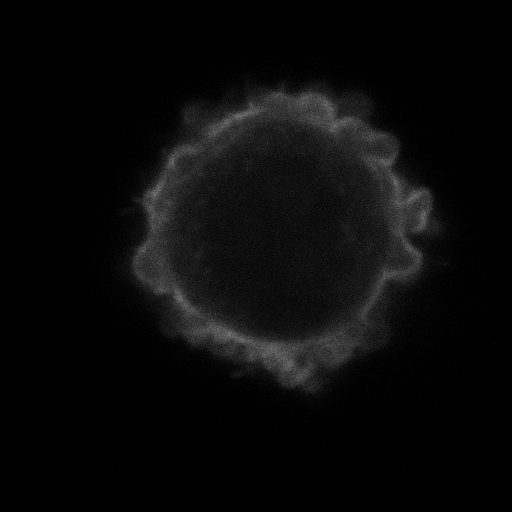

Supplement: Supplementary file 10 — Source data Fig. 5 [file 44318_2026_703_MOESM10_ESM.zip › Fig5/5A/5A_KN93.tif]

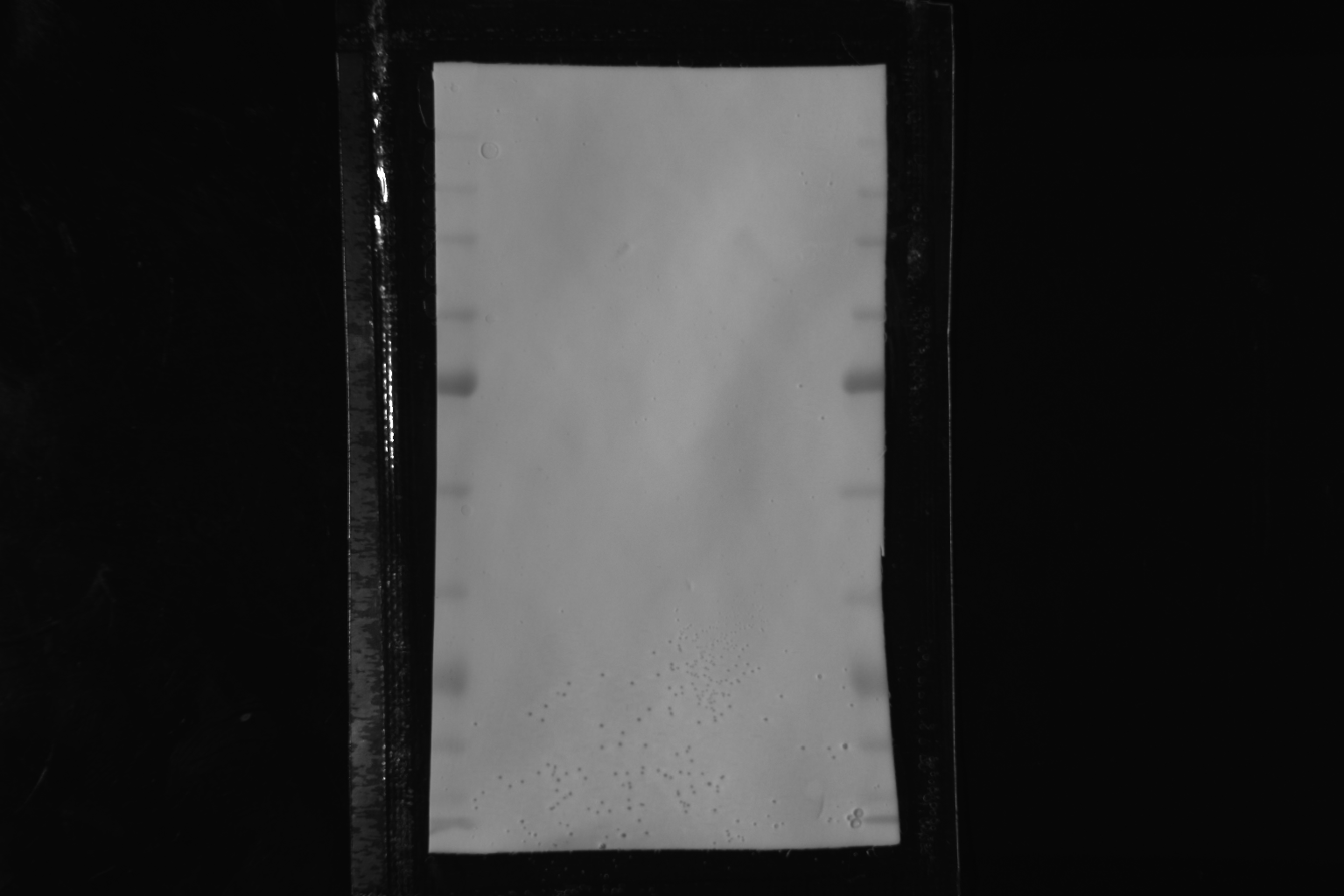

Supplement: Supplementary file 10 — Source data Fig. 5 [file 44318_2026_703_MOESM10_ESM.zip › Fig5/5D/uncropped WB/5D-1'_GFP_marker.tif]

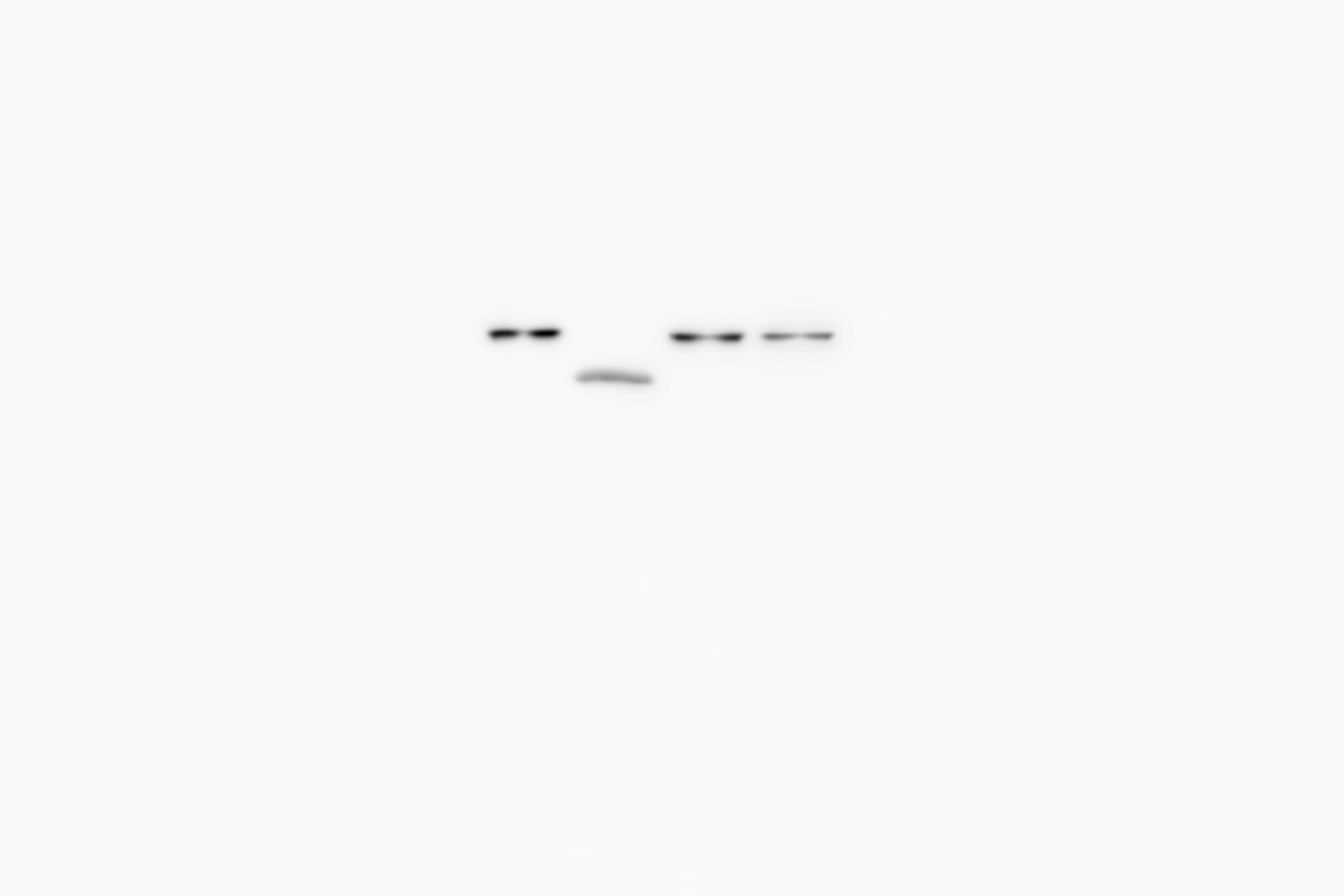

Supplement: Supplementary file 10 — Source data Fig. 5 [file 44318_2026_703_MOESM10_ESM.zip › Fig5/5D/uncropped WB/5D-1_GFP.tif]

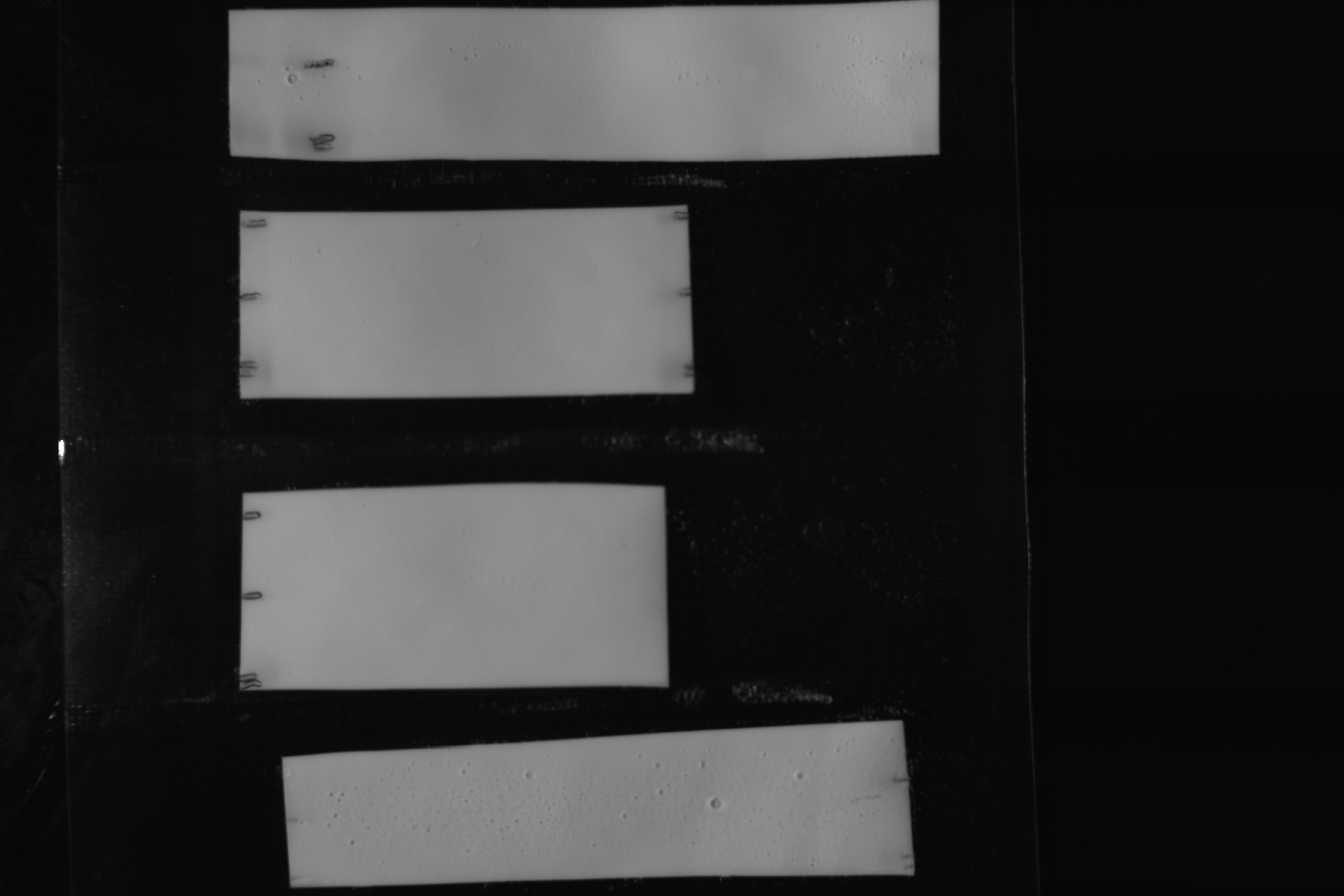

Supplement: Supplementary file 10 — Source data Fig. 5 [file 44318_2026_703_MOESM10_ESM.zip › Fig5/5D/uncropped WB/5D-2'_α-tubulin_marker.tif]

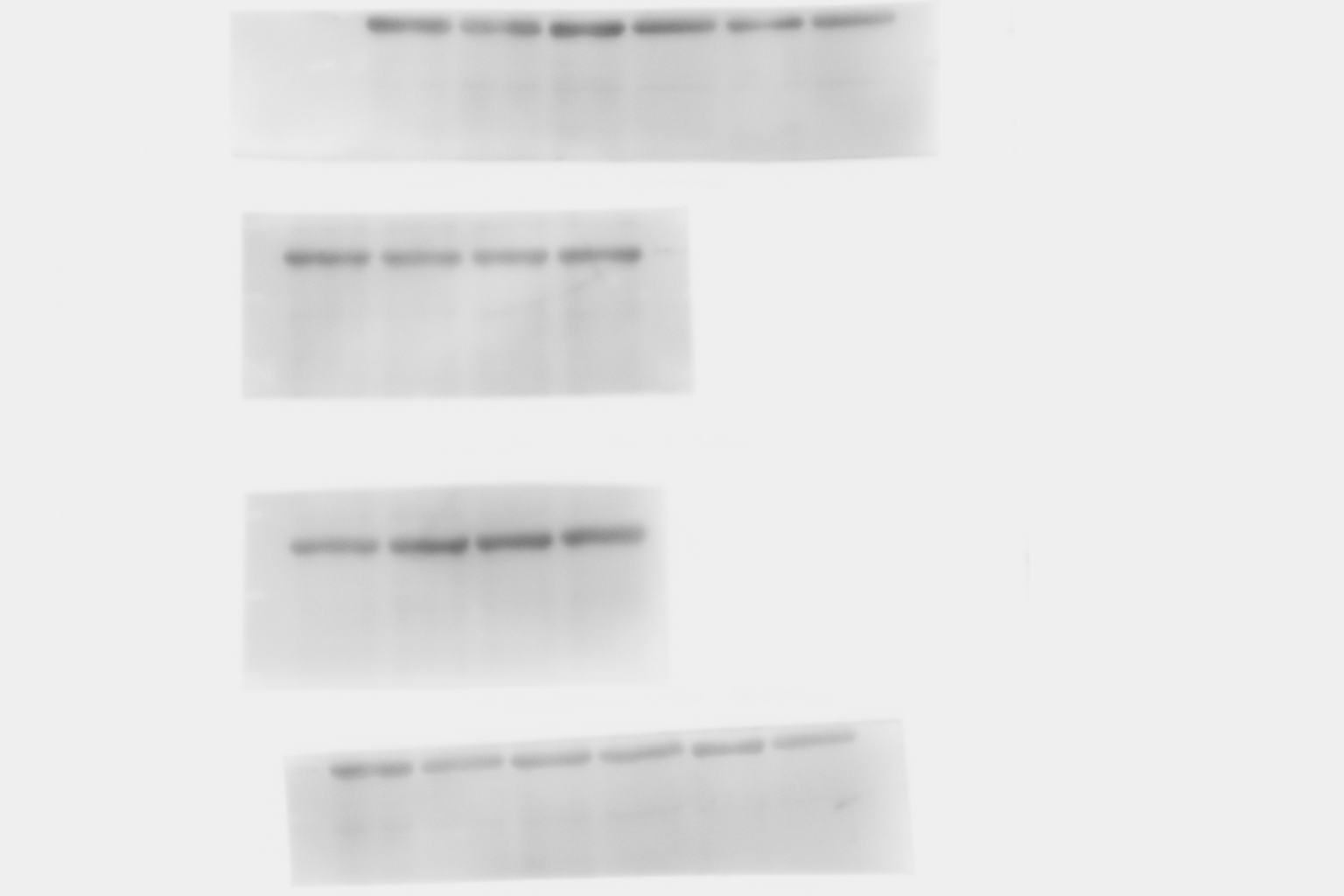

Supplement: Supplementary file 10 — Source data Fig. 5 [file 44318_2026_703_MOESM10_ESM.zip › Fig5/5D/uncropped WB/5D-2_α-tubulin.tif]

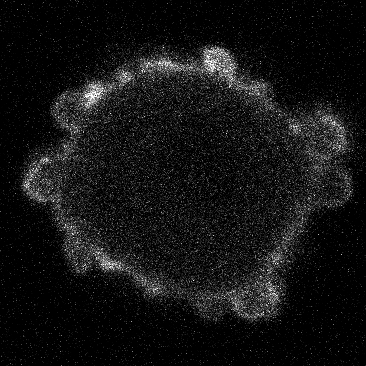

Supplement: Supplementary file 10 — Source data Fig. 5 [file 44318_2026_703_MOESM10_ESM.zip › Fig5/5E/5E_GFP-CaMKIIγ 2TD_Lifeact.tif]

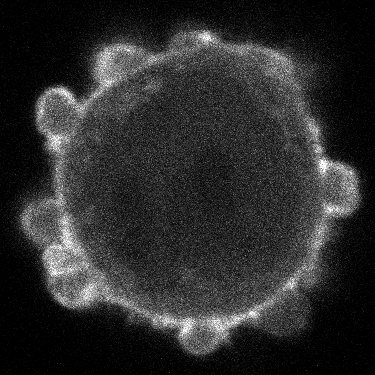

Supplement: Supplementary file 10 — Source data Fig. 5 [file 44318_2026_703_MOESM10_ESM.zip › Fig5/5E/5E_GFP-CaMKIIγ deltaC_Lifeact.tif]

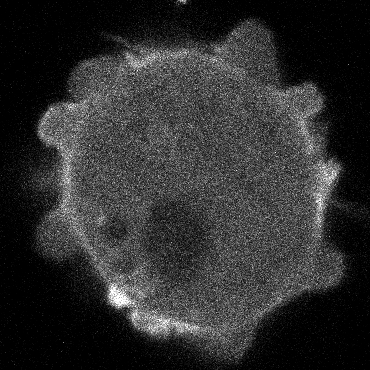

Supplement: Supplementary file 10 — Source data Fig. 5 [file 44318_2026_703_MOESM10_ESM.zip › Fig5/5E/5E_GFP-CaMKIIγ I205K_Lifeact.tif]

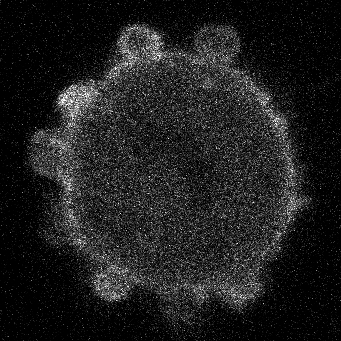

Supplement: Supplementary file 11 — Source data Fig. 6 [file 44318_2026_703_MOESM11_ESM.zip › Fig6/6A/6A_DKO_GFP-Mena_Lifeact.tif]

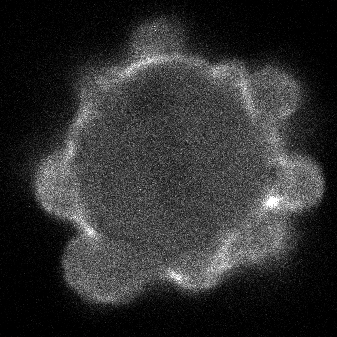

Supplement: Supplementary file 11 — Source data Fig. 6 [file 44318_2026_703_MOESM11_ESM.zip › Fig6/6A/6A_WT_GFP-Mena_Lifeact.tif]

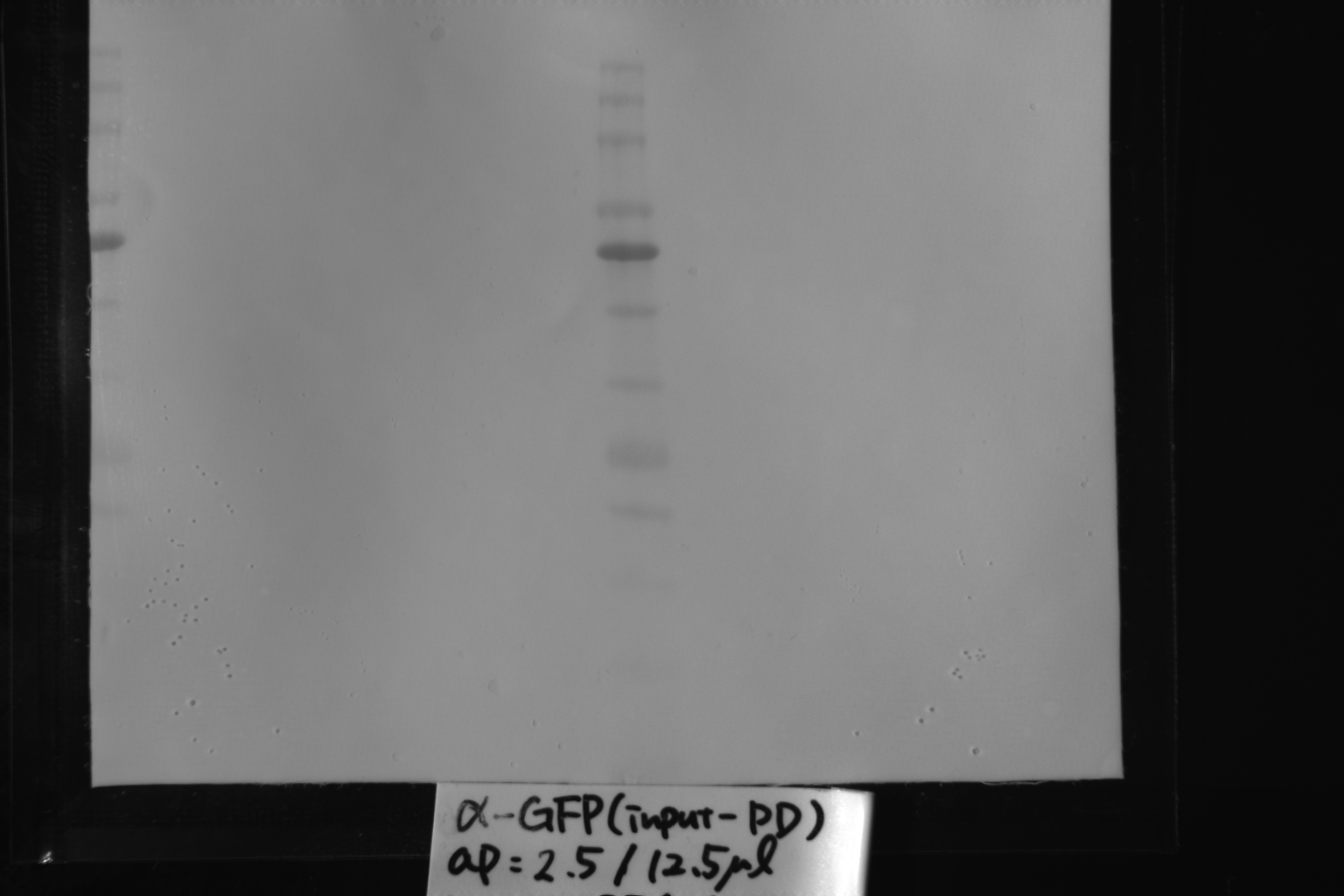

Supplement: Supplementary file 11 — Source data Fig. 6 [file 44318_2026_703_MOESM11_ESM.zip › Fig6/6D/uncropped WB/6D-1'_WB GFP_marker.tif]

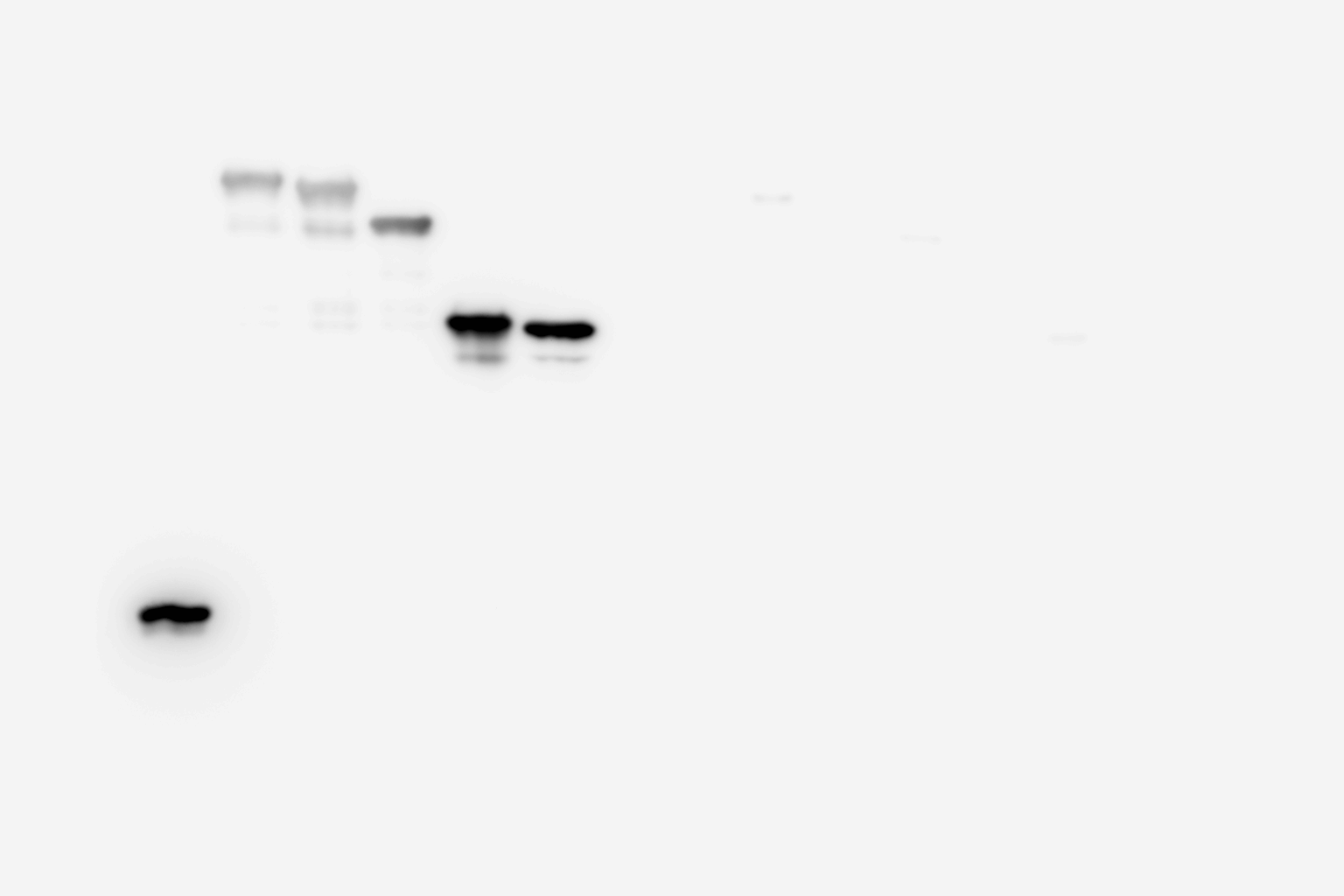

Supplement: Supplementary file 11 — Source data Fig. 6 [file 44318_2026_703_MOESM11_ESM.zip › Fig6/6D/uncropped WB/6D-1-1_WB GFP.tif]

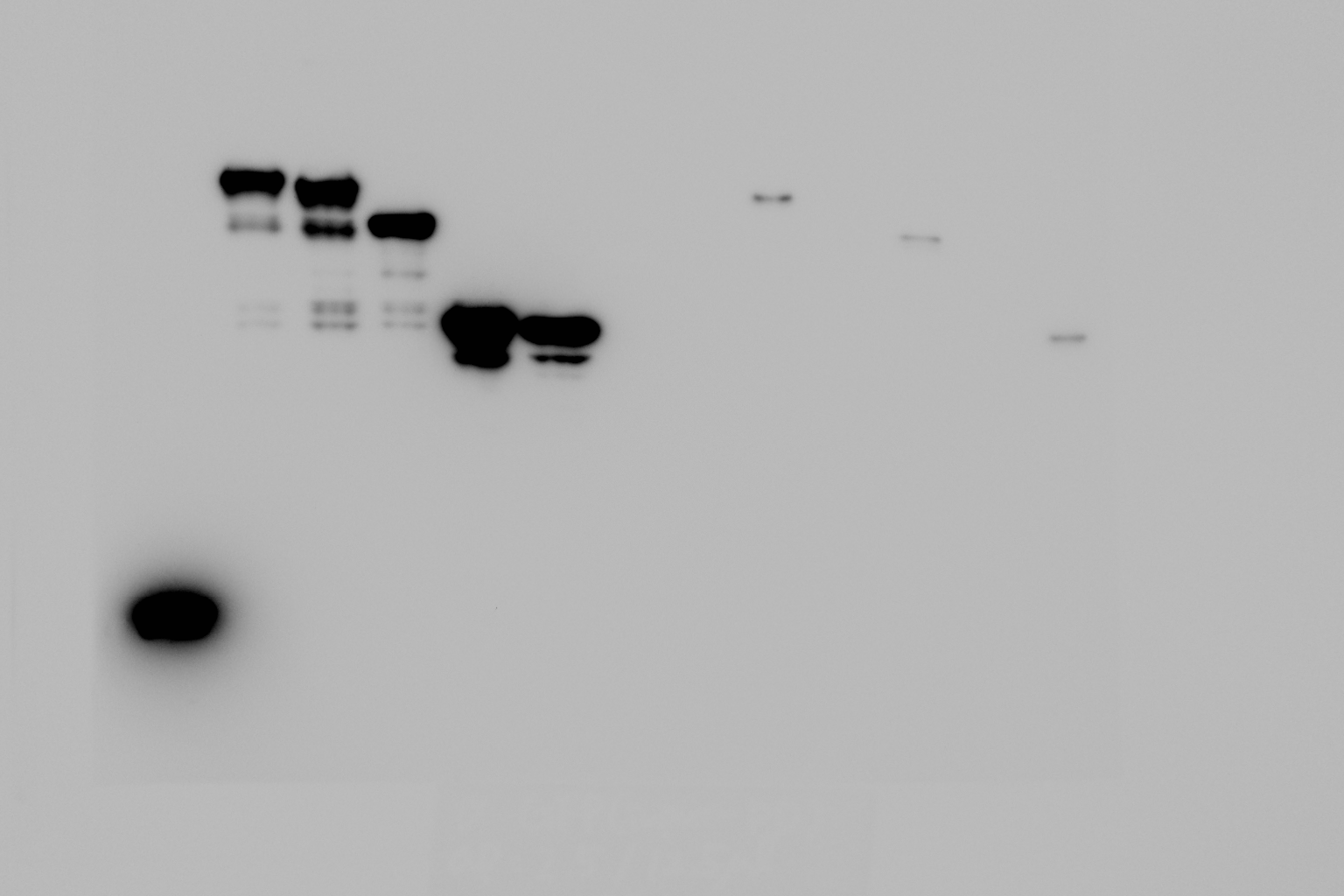

Supplement: Supplementary file 11 — Source data Fig. 6 [file 44318_2026_703_MOESM11_ESM.zip › Fig6/6D/uncropped WB/6D-1-2_WB GFP.tif]

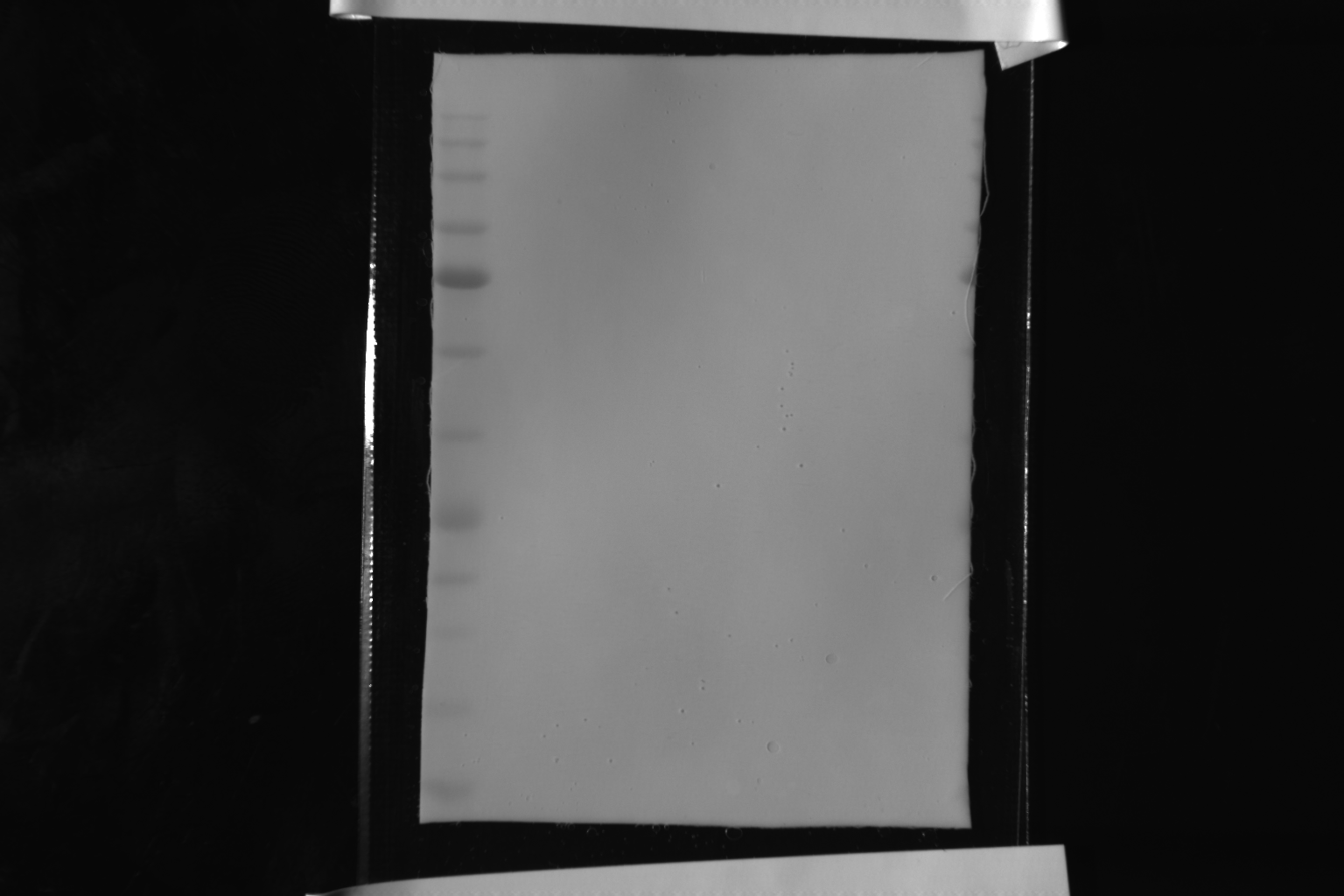

Supplement: Supplementary file 11 — Source data Fig. 6 [file 44318_2026_703_MOESM11_ESM.zip › Fig6/6D/uncropped WB/6D-2'_WB GST_marker.tif]

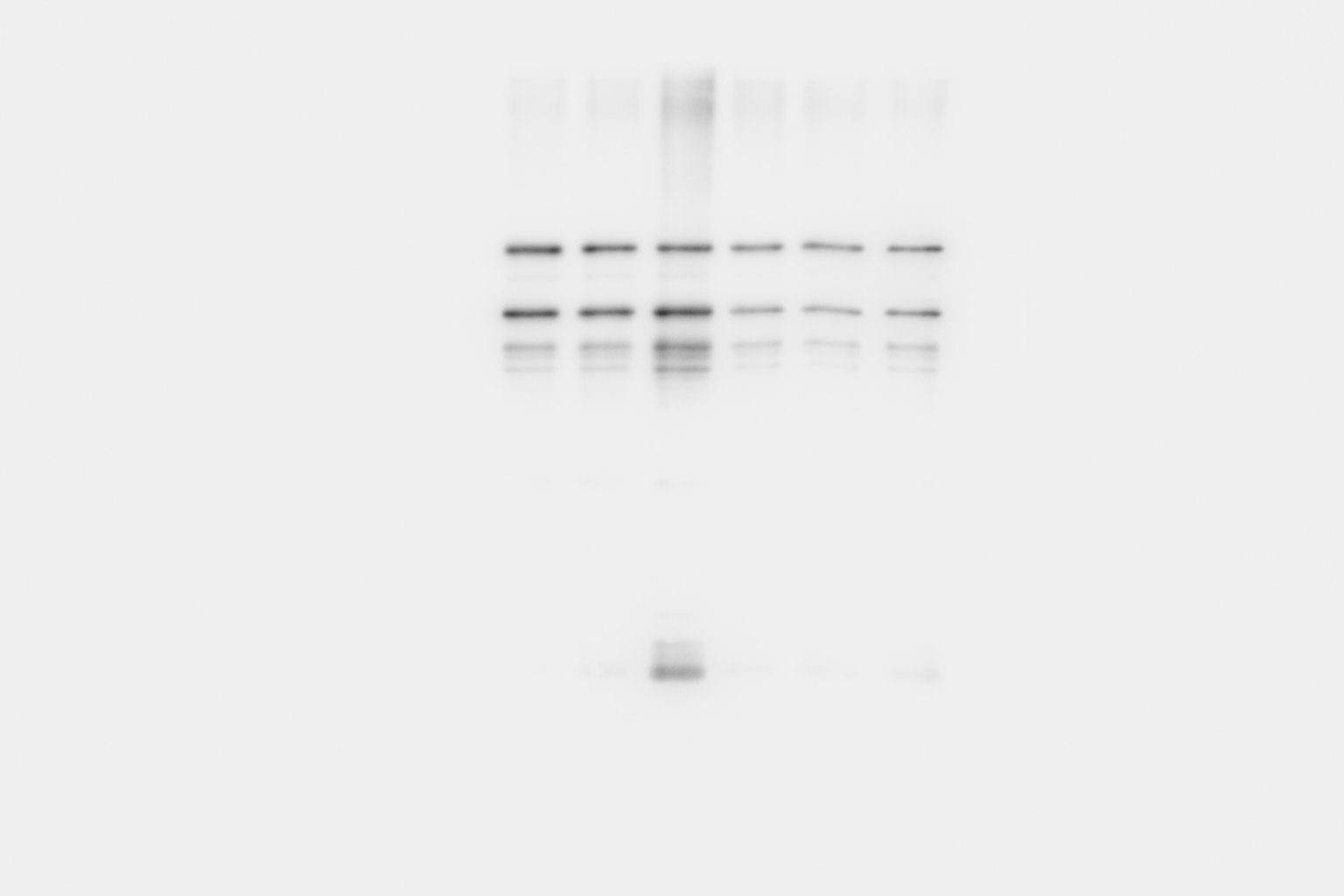

Supplement: Supplementary file 11 — Source data Fig. 6 [file 44318_2026_703_MOESM11_ESM.zip › Fig6/6D/uncropped WB/6D-2_WB GST.tif]

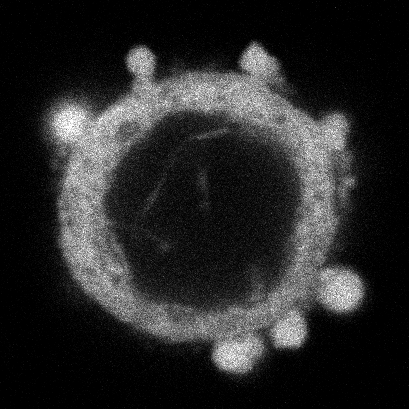

Supplement: Supplementary file 11 — Source data Fig. 6 [file 44318_2026_703_MOESM11_ESM.zip › Fig6/6E/6E_GFP-Mena_RFP.tif]

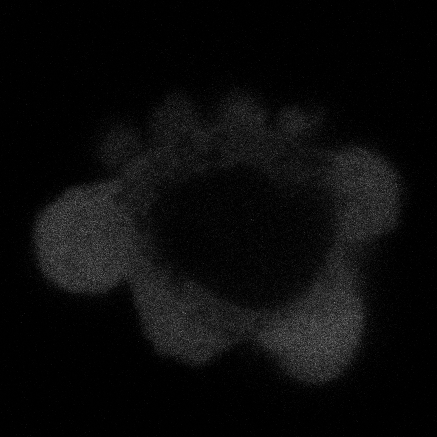

Supplement: Supplementary file 11 — Source data Fig. 6 [file 44318_2026_703_MOESM11_ESM.zip › Fig6/6E/6E_GFP-Mena_scarlet-CaMKIIγ T287A.tif]

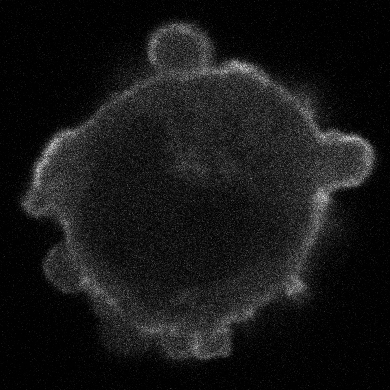

Supplement: Supplementary file 11 — Source data Fig. 6 [file 44318_2026_703_MOESM11_ESM.zip › Fig6/6G/6G_DKO_GFP-ERK1_Lifeact.tif]

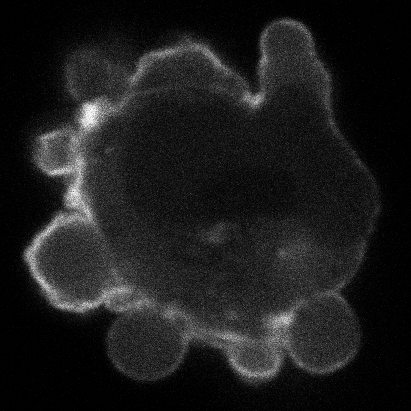

Supplement: Supplementary file 11 — Source data Fig. 6 [file 44318_2026_703_MOESM11_ESM.zip › Fig6/6G/6G_WT_GFP-ERK1_Lifeact.tif]
