# Supplementary material for: CaMKII nucleates an osmotic protein supercomplex to induce cellular bleb expansion
Source: EMBO J. 2026 Feb 3;45(8):2433–55. doi: 10.1038/s44318-026-00703-5 (PMC13083957; doi:10.1038/s44318-026-00703-5)
Supplement: Supplementary file 7 — Source data Fig. 2 [file 44318_2026_703_MOESM7_ESM.zip › Fig2/2A/2A_WB annotation.pptx]

## Slide 1
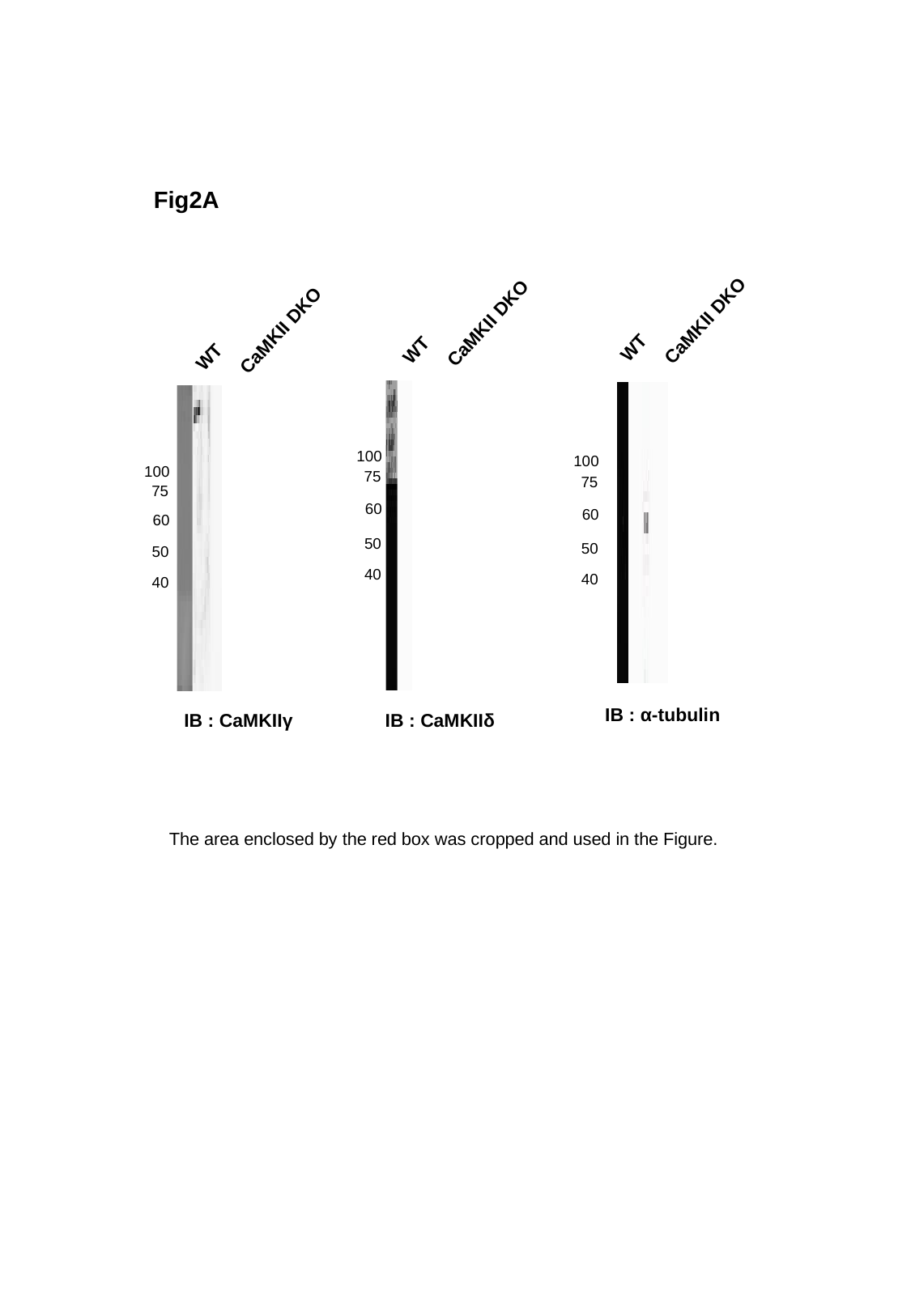

Fig2A
CaMKII DKO
CaMKII DKO
CaMKII DKO
WT
WT
WT
100
75
60
50
40
IB : α-tubulin
100
75
60
50
40
100
75
60
50
40
IB : CaMKIIγ
IB : CaMKIIδ
The area enclosed by the red box was cropped and used in the Figure.
