# Supplementary material for: CaMKII nucleates an osmotic protein supercomplex to induce cellular bleb expansion
Source: EMBO J. 2026 Feb 3;45(8):2433–55. doi: 10.1038/s44318-026-00703-5 (PMC13083957; doi:10.1038/s44318-026-00703-5)
Supplement: Supplementary file 8 — Source data Fig. 3 [file 44318_2026_703_MOESM8_ESM.zip › Fig3/3B/3B_WB annotation.pptx]

## Slide 1
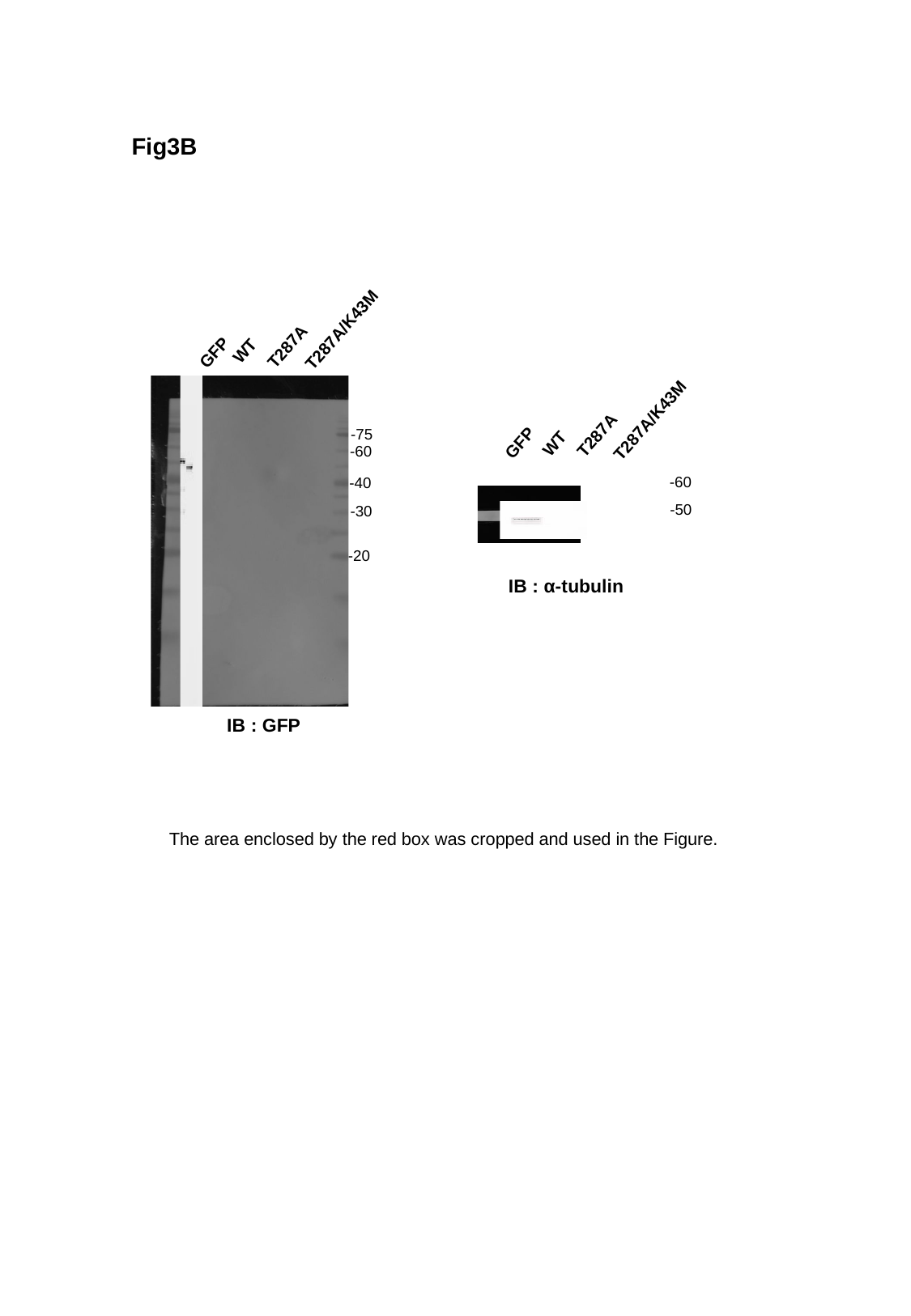

Fig3B
WT
GFP
T287A
T287A/K43M
WT
T287A
GFP
T287A/K43M
-75
-60
-60
-40
-50
-30
-20
IB : α-tubulin
IB : GFP
The area enclosed by the red box was cropped and used in the Figure.
