# Supplementary material for: CaMKII nucleates an osmotic protein supercomplex to induce cellular bleb expansion
Source: EMBO J. 2026 Feb 3;45(8):2433–55. doi: 10.1038/s44318-026-00703-5 (PMC13083957; doi:10.1038/s44318-026-00703-5)
Supplement: Supplementary file 10 — Source data Fig. 5 [file 44318_2026_703_MOESM10_ESM.zip › Fig5/5D/5D_WB annotation.pptx]

## Slide 1
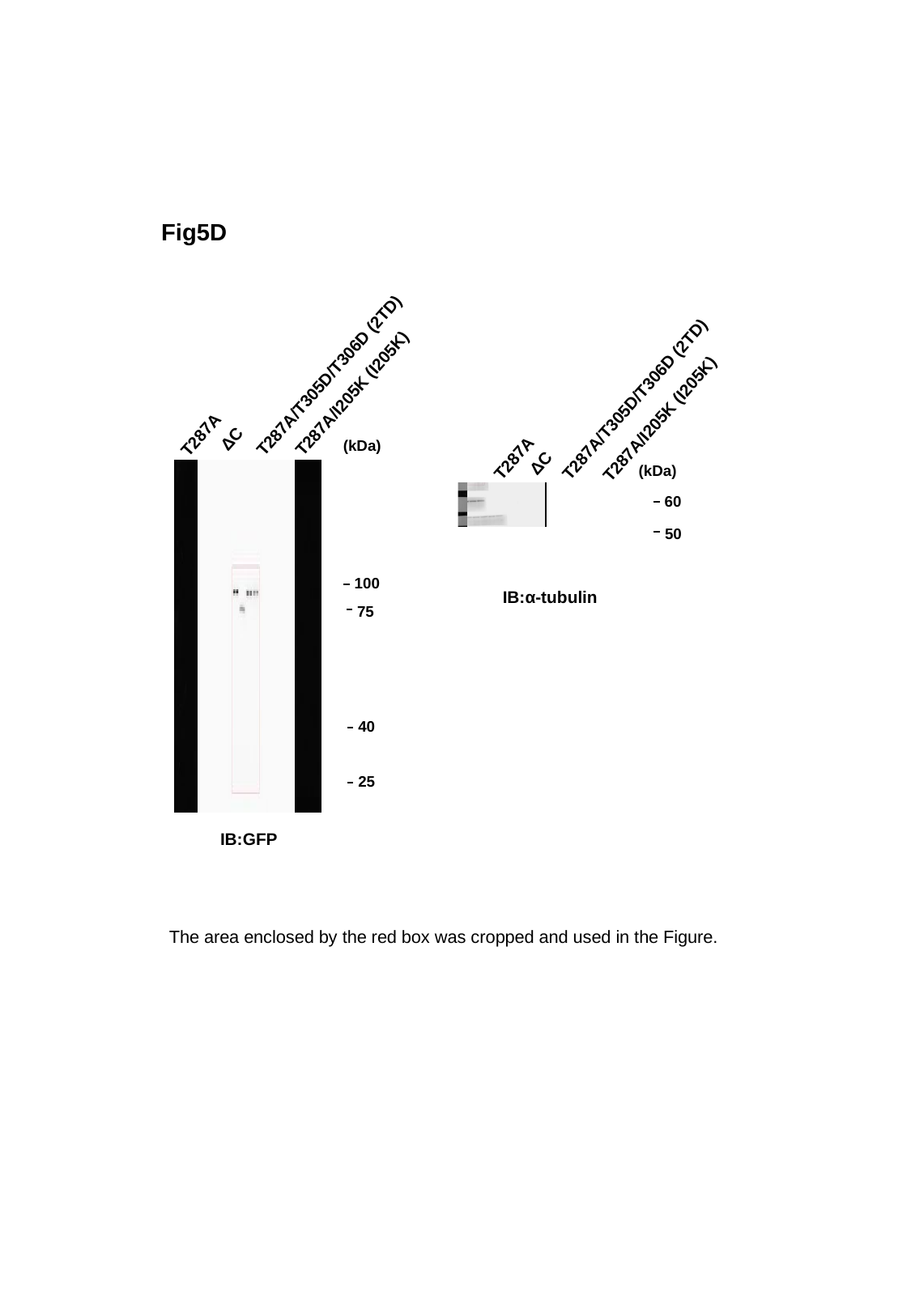

Fig5D
T287A/T305D/T306D (2TD)
T287A/I205K (I205K)
T287A/T305D/T306D (2TD)
ΔC
T287A
T287A/I205K (I205K)
ΔC
T287A
(kDa)
(kDa)
IB:α-tubulin
60
50
100
75
40
25
IB:GFP
The area enclosed by the red box was cropped and used in the Figure.
