# Supplementary material for: CaMKII nucleates an osmotic protein supercomplex to induce cellular bleb expansion
Source: EMBO J. 2026 Feb 3;45(8):2433–55. doi: 10.1038/s44318-026-00703-5 (PMC13083957; doi:10.1038/s44318-026-00703-5)
Supplement: Supplementary file 11 — Source data Fig. 6 [file 44318_2026_703_MOESM11_ESM.zip › Fig6/6D/6D_WB annotation.pptx]

## Slide 1
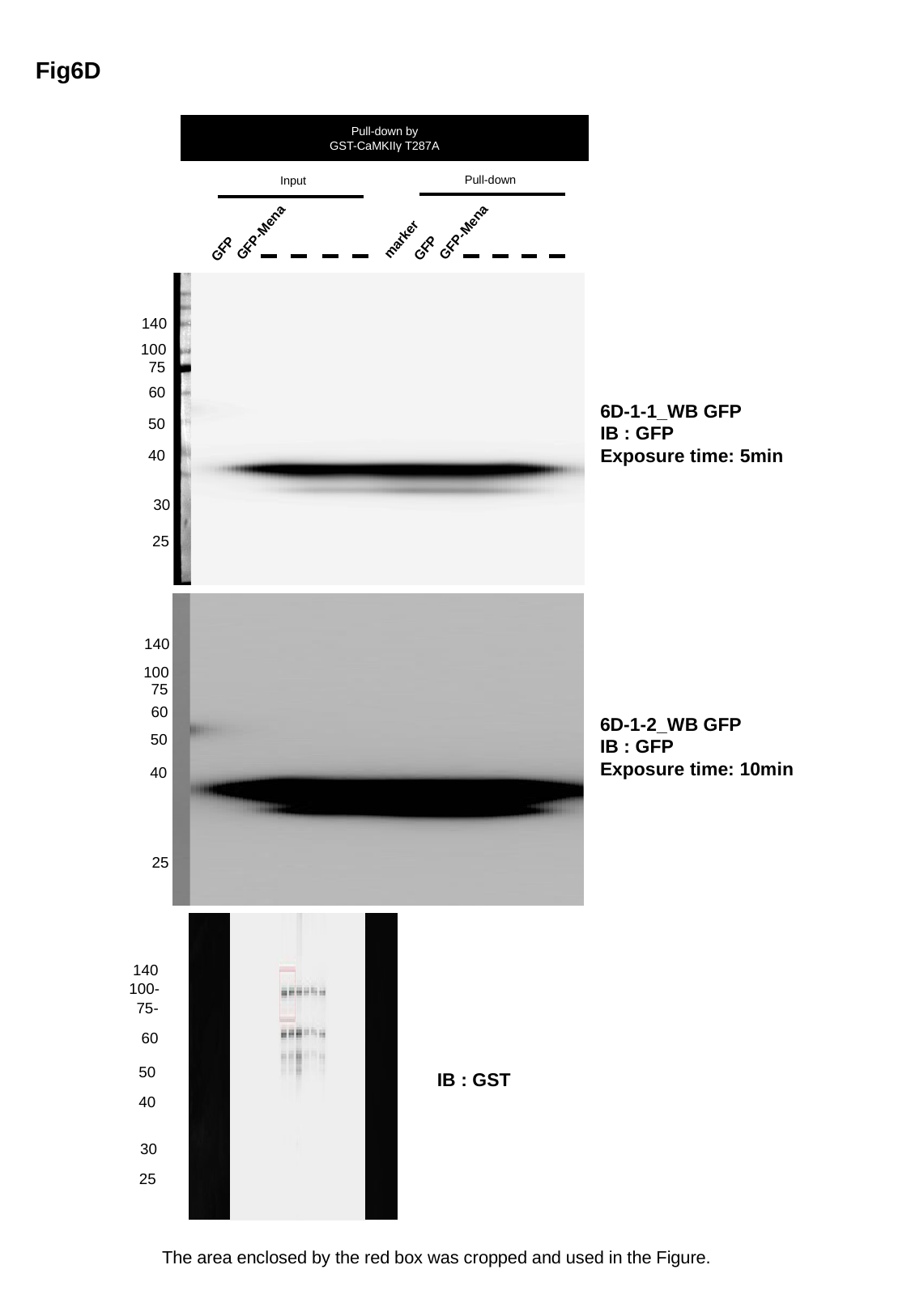

Fig6D
Pull-down by
GST-CaMKIIγ T287A
Pull-down
Input
marker
GFP-Mena
GFP-Mena
GFP
GFP
140
100
75
60
50
40
25
6D-1-1_WB GFP
IB : GFP
Exposure time: 5min
30
140
100
75
60
50
40
25
6D-1-2_WB GFP
IB : GFP
Exposure time: 10min
140
100-
75-
60
50
IB : GST
40
30
25
The area enclosed by the red box was cropped and used in the Figure.
