# Supplementary material for: CaMKII nucleates an osmotic protein supercomplex to induce cellular bleb expansion
Source: EMBO J. 2026 Feb 3;45(8):2433–55. doi: 10.1038/s44318-026-00703-5 (PMC13083957; doi:10.1038/s44318-026-00703-5)
Supplement: Supplementary file 14 — Expanded View Figures [file 44318_2026_703_MOESM14_ESM.pdf]

Expanded View Figures

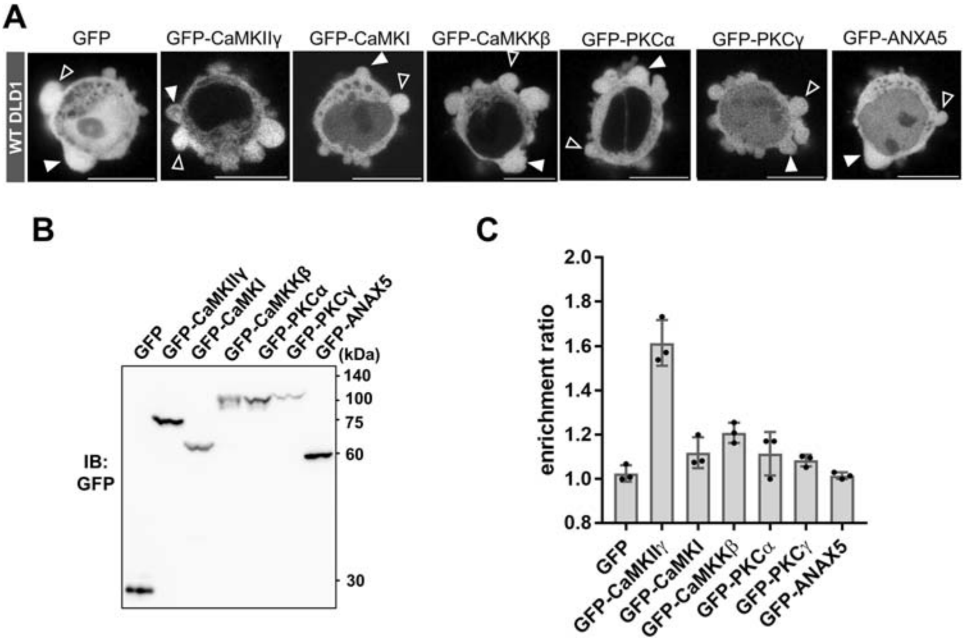

Figure EV1. Screening for cytoplasmic Ca^{2+}-related proteins based on their enrichment within blebs.

(**A**) Wild-type DLD1 cells expressing GFP-tagged cytoplasmic proteins subjected to screening. Black arrowheads indicate expanding blebs, and white arrowheads indicate retracting blebs (Scale bar: 10 µm). (**B**) Western blot analysis of HEK293 cell lysates expressing the vectors used for screening, probed with an anti-GFP antibody. (**C**) Maximum enrichment ratio of each GFP-tagged protein relative to monomeric RFP within expanding blebs. Quantification was performed for three blebs per protein and results are shown as mean of three biological replicates ± SD.

 © The Author(s)

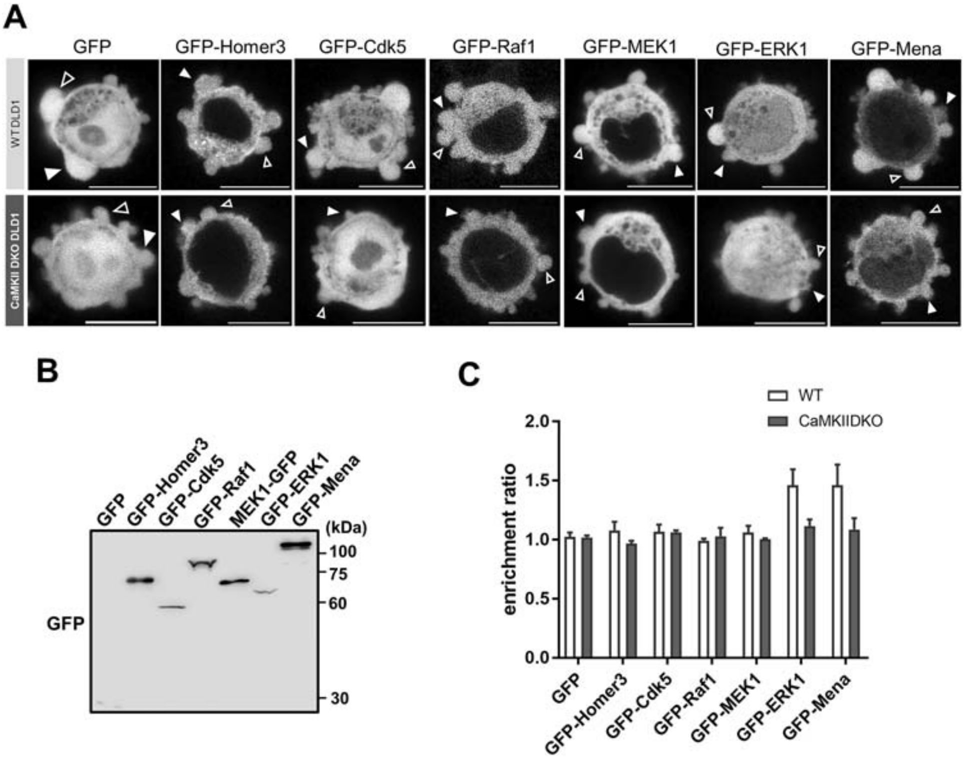

Figure EV2. Analysis of bleb accumulation of GFP-tagged CaMKII-related cytoplasmic proteins.

(**A**) Wild-type DLD1 cells expressing GFP-tagged cytoplasmic proteins reported to interact with CaMKII. Black arrowheads indicate expanding blebs, and white arrowheads indicate retracting blebs (Scale bar: 10 µm). (**B**) Western blot analysis of HEK293 cell lysates expressing the vectors used for localization analysis, probed with an anti-GFP antibody. (**C**) Quantification of the maximum enrichment ratio of each GFP-tagged protein relative to monomeric RFP within expanding blebs. Five blebs were analyzed for each protein and results are shown as mean of three biological replicates ± SD.

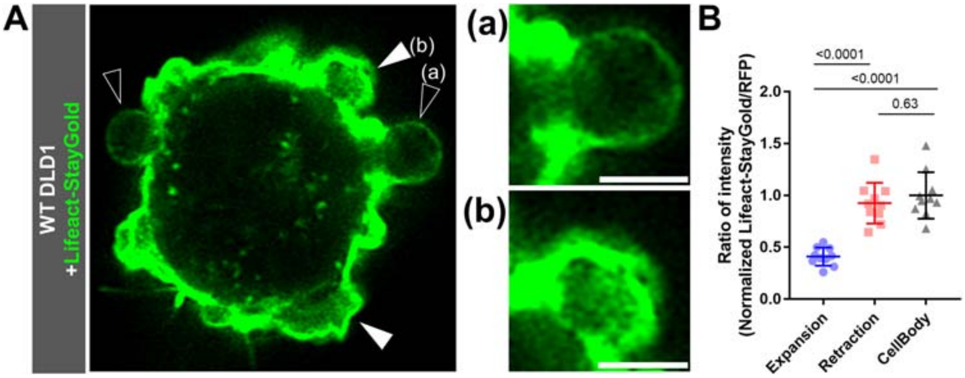

Figure EV3. Super-resolution imaging of the cytoplasmic actin network during bleb expansion and retraction.

(**A**) Super-resolution images of WT cells expressing Lifeact-StayGold, acquired with Airyscan2 and processed by Airyscan processing. Black arrowheads indicate expanding blebs, while white arrowheads indicate retracting blebs (Scale bar: 10 µm). Panels show (a) an expanding bleb, (b) a retracting bleb (Scale bar: 2 µm). Notably, differences in actin organization are observed not only at the cortex but also within the cytoplasm. (**B**) WT cells co-expressing Lifeact-StayGold and RFP were imaged using Airyscan2, and super-resolution images obtained after Airyscan processing were used for analysis. For each bleb, the mean fluorescence intensities of Lifeact-StayGold and RFP were measured in three cytoplasmic regions: the interior of expanding blebs, the interior of retracting blebs, and the cell-body cytoplasm adjacent to the bleb. The ratio of Lifeact-StayGold to RFP intensity was calculated for each region. Quantification was performed on 10 blebs, followed by one-way ANOVA with Tukey's multiple comparisons test; p values are $p < 0.0001$ (for Expansion vs. Retraction), $p < 0.0001$ (for Expansion vs. CellBody), $p = 0.6293$ (for Retraction vs. CellBody).

 © The Author(s)

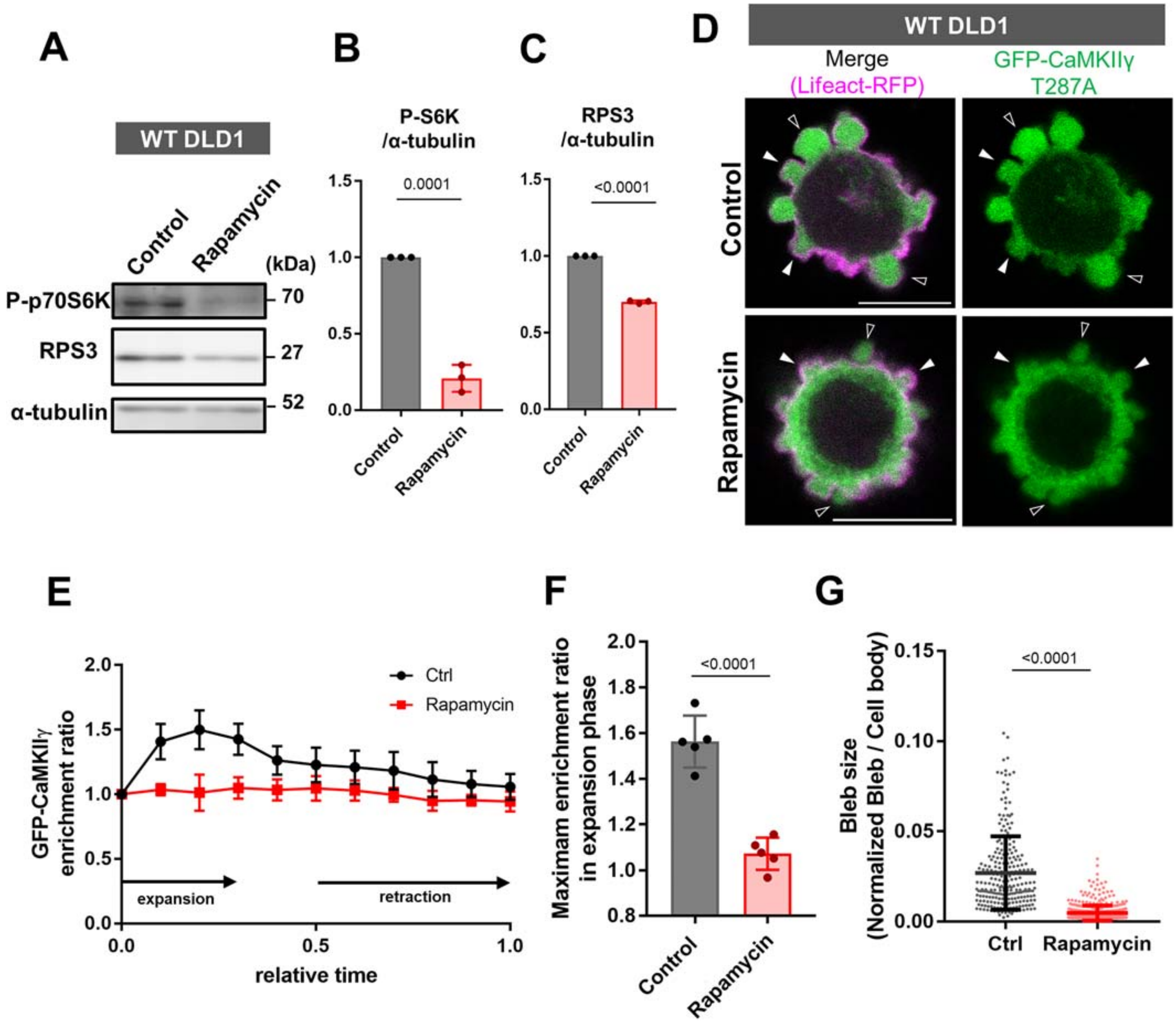

Figure EV4. Effects of rapamycin-induced ribosome depletion on CaMKII dynamics and bleb morphology.

(**A**) Western blot analysis of lysates from wild-type DLD1 cells treated with the indicated reagents. α-Tubulin was used as a loading control. (**B**) Quantification of the Western blot results. A marked decrease in phospho-p70S6K levels was observed in rapamycin-treated cells, confirming effective inhibition of mTORC1. Data represents the mean ± SD from three independent experiments. Statistical significance was Student's t-test; p value is $p = 0.0001$. (**C**) Quantification of the Western blot results. Rapamycin-treated cells showed a significant reduction in RPS3 signal intensity. Each experiment was performed three times. Data represents the mean ± SD from three independent experiments. Statistical significance was Student's t-test; p value is $p < 0.0001$. (**D**) Fluorescence microscopy images of DLD1 cells expressing GFP–CaMKII and Lifeact–RFP after treatment with the indicated reagents. Black arrowheads indicate expanding blebs, while white arrowheads indicate retracting blebs. In cells treated with rapamycin, accumulation of GFP–CaMKII within expanding blebs was markedly reduced, accompanied by a decrease in bleb size. (**E**) Temporal changes in CaMKII enrichment levels within blebs of cells treated with various reagents. Each dataset represents quantification of five blebs, and the mean ± SD is plotted for each time point. (**F**) Maximum enrichment ratio of GFP–CaMKII relative to monomeric RFP within expanding blebs of cells treated with the indicated reagents. Quantification was performed for five blebs per group, and statistical significance was assessed using Student's t-test; p value is $p < 0.0001$. (**G**) Distribution of bleb sizes in WT cells treated with indicated agents. The areas of all blebs in 20 cells were quantified. Results are shown as mean of four biological replicates ± SD and the p values of Student's t-test are indicated; p value is $p < 0.0001$.

© The Author(s)
